# l-2-Hydroxyglutarate remodeling of the epigenome and epitranscriptome creates a metabolic vulnerability in kidney cancer models

**DOI:** 10.1172/JCI171294

**Published:** 2024-05-14

**Authors:** Anirban Kundu, Garrett J. Brinkley, Hyeyoung Nam, Suman Karki, Richard Kirkman, Madhuparna Pandit, EunHee Shim, Hayley Widden, Juan Liu, Yasaman Heidarian, Nader H. Mahmoudzadeh, Alexander J. Fitt, Devin Absher, Han-Fei Ding, David K. Crossman, William J. Placzek, Jason W. Locasale, Dinesh Rakheja, Jonathan E. McConathy, Rekha Ramachandran, Sejong Bae, Jason M. Tennessen, Sunil Sudarshan

**Affiliations:** 1 Department of Urology, University of Alabama at Birmingham, Birmingham, Alabama, USA.; 2 Department of Urology, University of Arizona, Tuscon, Arizona, USA.; 3 Department of Biochemistry and Molecular Genetics, University of Alabama at Birmingham, Birmingham, Alabama, USA.; 4 Department of Pharmacology and Cancer Biology, Duke University School of Medicine, Durham, North Carolina, USA.; 5 Department of Biology, Indiana University, Bloomington, Indiana, USA.; 6 HudsonAlpha Institute for Biotechnology, Huntsville, Alabama, USA.; 7 Department of Pathology and; 8 Department of Genetics, University of Alabama at Birmingham, Birmingham, Alabama, USA.; 9 Department of Pathology, University of Texas Southwestern Medical Center, Dallas, Texas, USA.; 10 Department of Radiology and; 11 Department of Medicine, University of Alabama at Birmingham, Birmingham, Alabama, USA.

**Keywords:** Metabolism, Oncology, Urology

## Abstract

Tumor cells are known to undergo considerable metabolic reprogramming to meet their unique demands and drive tumor growth. At the same time, this reprogramming may come at a cost with resultant metabolic vulnerabilities. The small molecule l-2-hydroxyglutarate (l-2HG) is elevated in the most common histology of renal cancer. Similarly to other oncometabolites, l-2HG has the potential to profoundly impact gene expression. Here, we demonstrate that l-2HG remodels amino acid metabolism in renal cancer cells through combined effects on histone methylation and RNA *N*^6^-methyladenosine. The combined effects of l-2HG result in a metabolic liability that renders tumors cells reliant on exogenous serine to support proliferation, redox homeostasis, and tumor growth. In concert with these data, high–l-2HG kidney cancers demonstrate reduced expression of multiple serine biosynthetic enzymes. Collectively, our data indicate that high–l-2HG renal tumors could be specifically targeted by strategies that limit serine availability to tumors.

## Abstract 2

The oncometabolite ʟ-2-hydroxyglutarate suppresses expression of serine biosynthetic enzymes in renal cancer.

## Graphical abstract

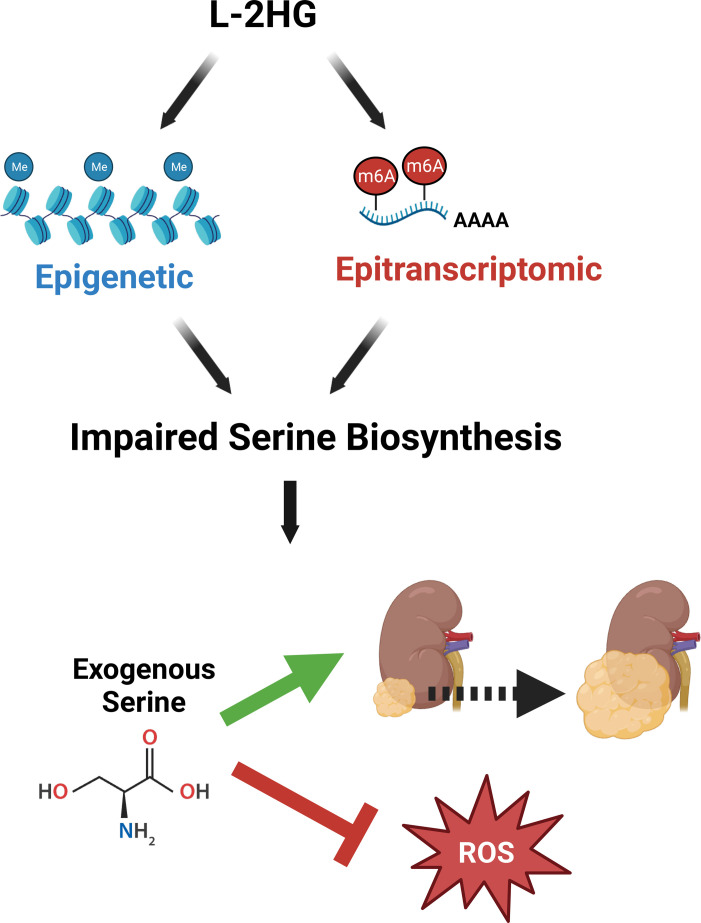

## Introduction

Oncometabolites are small molecules that aberrantly accumulate within tumor cells. These small molecules can have either tumor-promoting or -suppressive effects, indicating that their impact on tumor biology is highly context dependent. Oncometabolites have been identified in multiple subtypes of renal cell carcinoma (RCC), including fumarate and succinate, which are due to loss-of-function mutations in the genes encoding fumarate hydratase (*FH*) and succinate dehydrogenase (*SDHB*, *SDHC*, *SDHD*), respectively (1). Elevations of d-2-hydroxyglutarate have been identified in other tumor types in the context of isocitrate dehydrogenase (*IDH1/2*) mutations (2–4). We previously reported elevations of l-2-hydroxyglutarate (l-2HG) in the most common histology of kidney cancer, referred to as clear cell renal cell carcinoma (ccRCC) (5). A shared feature of the oncometabolites described to date is their structural similarity with α-ketoglutarate (α-KG). α-KG is a cofactor for enzymes that catalyze both histone and nucleotide modifications. Prior studies have indicated that the biologically relevant target(s) of oncometabolites are enzymes that require α-KG, either as a substrate or as a cofactor (6–11). Deciphering the relevant target(s) of an oncometabolite and the ensuing impact on tumor biology could inform novel treatment strategies.

l-2HG accumulates in RCC due to loss of the enzyme l-2HG dehydrogenase (L2HGDH). We previously identified that approximately 25% of primary ccRCCs have excessively high l-2HG levels (≥20-fold elevation) with further increases in metastatic tissue deposits (5, 12). l-2HG promotes tumor growth in xenograft models. These data provide a compelling rationale to delineate l-2HG’s target(s) in RCC, which could inform novel approaches to treat l-2HG–driven tumors. We therefore undertook unbiased approaches to study l-2HG’s impact on tumor biology. Our studies identified that l-2HG remodels tumor metabolism through combined effects on both the epigenome and the epitranscriptome. These effects lead to a metabolic liability resulting in dependence on exogenous serine to support proliferation, redox homeostasis, and tumor growth. While strategies to target serine availability have been proposed, a major gap in our knowledge is which tumors are most likely to respond to such strategies, and insight into the mechanisms that promote this sensitivity. Our data reveal a potential oncometabolic biomarker in elevated l-2HG that could inform precision-based approaches that target metabolism for the most common variant of kidney cancer.

## Results

### l-2HG suppresses mRNA expression of amino acid metabolism genes.

**Figure 1:**
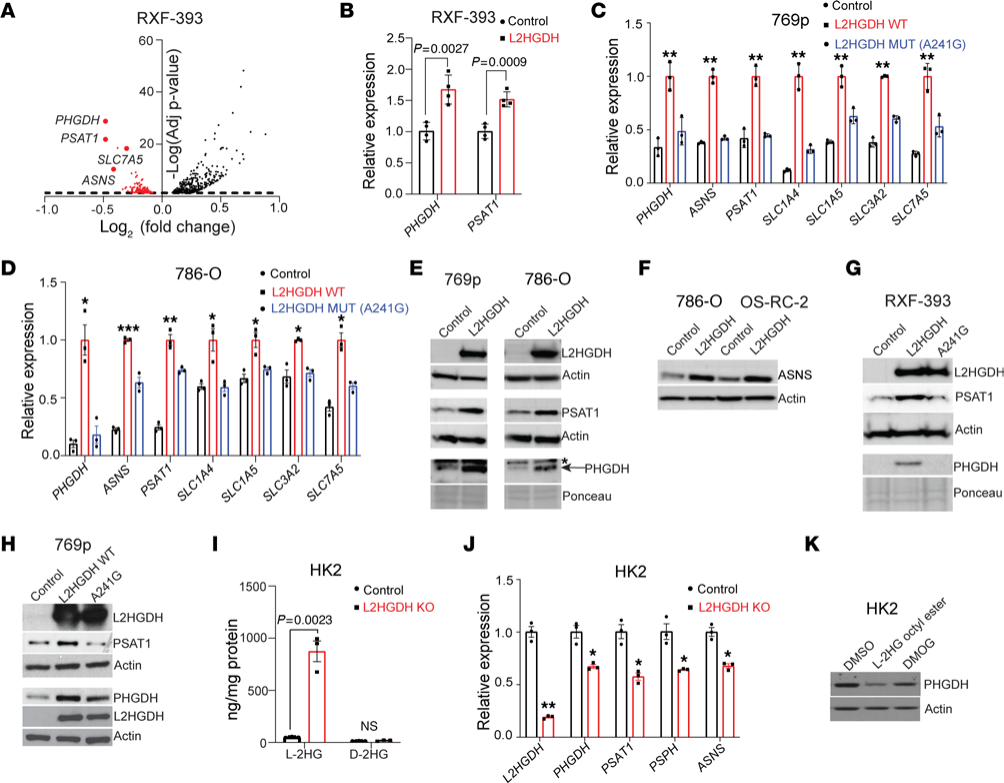
High l-2HG suppresses amino acid synthesis and transporter genes in RCC. (**A**) Differentially expressed transcripts in RXF-393 control vector (high l-2HG) relative to cells transduced with L2HGDH cDNA (low l-2HG). Amino acid synthesis and transporter genes are indicated (horizontal dashed line denotes *P* value of 0.05). (**B**) Relative mRNA of *PHGDH* and *PSAT1* (normalized to RPLPO) from RXF-393 cells stably expressing control black vector or L2HGDH (red). Data are shown as mean ± SD from *n =* 4 biological replicates. (**C** and **D**) Relative mRNA levels of amino acid synthetic/transporter genes from 769p (**C**) and 786-O (**D**) cells stably expressing the indicated construct. Expression was normalized to RPLPO. Data are shown as mean ± SD from *n =* 4 biological replicates. *P < 0.05, **P < 0.005, ***P < 0.0001. (**E**) Immunoblot of PHGDH, PSAT1, and L2HGDH protein from 769p and 786-O cells transduced with control vector or L2HGDH cDNA. Actin (β-actin) or Ponceau S stain was used as loading control. Blots are from the same biological sample run contemporaneously. (**F**) Immunoblot of ASNS protein from 786-O and OS-RC-2 RCC cells transduced with control vector or L2HGDH cDNA. Actin was used as loading control. (**G** and **H**) Immunoblot of RXF-393 (**G**) and 769p (**H**) cells stably expressing control vector, L2HGDH (WT), or L2HGDH A241G (catalytic mutant). Actin was used as loading control. (**I**) Tandem MS analysis for l-2HG and D-2HG metabolites from control (black) or L2HGDH-KO (red) HK-2 renal epithelial cells normalized to protein content. Data are shown as mean ± SEM from *n =* 3 biological replicates. (**J**) mRNA expression of the indicated genes was examined by RT-qPCR from control (black) or L2H DH-KO (red) HK-2 cells. Data are expressed as mean ± SEM from *n =* 3 biological replicates. *P < 0.05, **P < 0.005. (**K**) Immunoblot for PHGDH protein from HK-2 cells treated with either DMSO or l-2HG octyl ester (5 mM) or DMOG (1 mM) for 4 hours. Actin was used as loading control.

To assess l-2HG’s impact on RCC biology in an unbiased manner, we performed RNA-Seq analysis of a paired cell line system. We previously noted that RXF-393 RCC cells have reduced expression of L2HGDH enzyme with a concomitant increase in cellular l-2HG levels (5). We stably transduced these cells with control vector and L2HGDH cDNA to perform a comparative analysis between high– and low–l-2HG cells. We observed that restoring L2HGDH (i.e., lowering l-2HG levels) resulted in the increased expression of mRNAs related to amino acid metabolism including biosynthetic enzymes and metabolite transporters (Figure 1A and Supplemental Table 1; supplemental material available online with this article; https://doi.org/10.1172/JCI171294DS1). Among the most significantly increased mRNAs upon L2HGDH restoration were those encoding phosphoglycerate dehydrogenase (*PHGDH*) and phosphoserine aminotransferase 1 (*PSAT1*). PHGDH and PSAT1 catalyze the first 2 of 3 steps of de novo serine biosynthesis. Accordingly, pathway analysis of RNA-Seq data demonstrated an enrichment of genes related to serine/glycine metabolism upon lowering of l-2HG levels (Supplemental Figure 1, A and B). We validated these data via real-time quantitative reverse transcriptase PCR (RT-qPCR) (Figure 1B). We next determined whether L2HGDH’s catalytic activity was required for this effect using a cDNA encoding a patient-derived mutant of L2HGDH (A241G). RCC cells transduced with WT L2HGDH demonstrated a prominent reduction in l-2HG levels, whereas the A241G mutant had only a modest effect on l-2HG levels (Supplemental Figure 1C). In multiple lines tested, we found that L2HGDH’s catalytic activity was required to increase mRNA expression of amino acid biosynthetic and transporter genes (Figure 1, C and D). We next determined the impact of restoring L2HGDH on protein levels of amino acid biosynthetic enzymes. In both 769p and 786-O RCC cells, restoring L2HGDH led to increased expression of both PHGDH and PSAT1 protein (Figure 1E). In addition, L2HGDH restoration led to increased levels of asparagine synthetase (ASNS) (Figure 1F). In alignment with the mRNA data, L2HGDH’s catalytic activity was required for the induction of PHGDH and PSAT1 protein levels in RCC cells (Figure 1, G and H). Based on these data, we used CRISPR/Cas9 to knock out L2HGDH expression in HK-2 immortalized renal epithelial cells, which have high L2HGDH enzyme expression and low basal l-2HG levels (5). *L2HGDH*-knockout cells demonstrated increased l-2HG levels without any significant increase in D-2HG (Figure 1I). Consistent with our gain-of-function studies, *L2HGDH* knockout led to reduced mRNA levels of amino acid biosynthetic genes (Figure 1J). Furthermore, rescue via transient transfection of knockout cells with L2HGDH cDNA led to increased PHGDH and PSAT1 protein (Supplemental Figure 1D). We next determined whether exogenous l-2HG could have similar effects. We treated cells with esterified l-2HG (octyl-ester), which raises intracellular levels of l-2HG (5). Consistent with our gain/loss-of-function studies with L2HGDH, exogenous l-2HG led to reduced expression of PHGDH (Figure 1K, compare first 2 lanes). Given these findings, we considered whether l-2HG’s effects could be through inhibition of α-KG–dependent dioxygenases. As an initial approach, we used dimethyloxalylglycine (DMOG). Owing to structural similarity to α-KG, DMOG can competitively inhibit α-KG–dependent dioxygenases. Similarly to exogenous l-2HG, DMOG treatment also reduced PHGDH expression in HK-2 renal epithelial cells (Figure 1K, last lane).

### l-2HG suppresses ATF4 expression.

**Figure 2:**
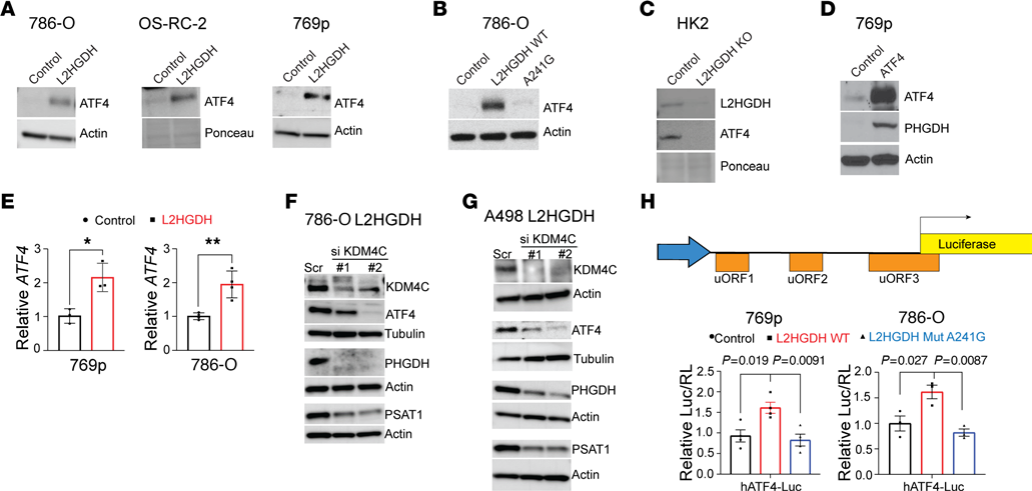
l-2HG suppression of ATF4. (**A**) Immunoblot for ATF4 protein from 786-O, OS-RC-2, and 769p RCC cells stably transduced with the indicated vectors. Actin was used as loading control. (**B**) Immunoblot for ATF4 protein from 786-O cells stably transduced with control vector, L2HGDH (WT), or L2HGDH A241G (mutant). Actin was used as loading control. (**C**) Immunoblot for L2HGDH and ATF4 protein from control and L2HGDH-KO HK-2 cells. Actin was used as loading control. (**D**) Immunoblot for ATF4 and PHGDH from 769p cells stably expressing either control vector or ATF4 cDNA. Actin was used as loading control. (**E**) Relative ATF4 mRNA normalized to TBP was examined by RT-qPCR from 769p and 786-O cells stably expressing either control (black) vector or L2HGDH cDNA (red). Data are expressed as mean ± SD from *n =* 3 (769p) or *n =* 4 (786-O) biological replicates. *P < 0.05, **P < 0.005. (**F** and **G**) 786-O (**F**) and A498 (**G**) cells stably transduced with L2HGDH cDNA were transiently transfected (55 hours) with either scramble siRNA (Scr) or siRNAs targeting KDM4C (#1, #2). Immunoblotting for KDM4C, ATF4, PHGDH, and PSAT1 was performed. Actin (β-actin) or tubulin (α-tubulin) was used as loading control. (**H**) Top: The construct containing the human ATF4 5′-uORFs preceding firefly luciferase. Bottom: Relative luciferase signal following transient transfection of the luciferase construct into 769p and 786-O cells stably expressing the indicated vector (control vector, black; L2HGDH WT, red; or L2HGDH A141G mutant, blue). Data were normalized to *Renilla* luciferase (Luc). Data are presented as mean ± SEM. ANOVA was used, and Tukey’s post hoc *P* values are shown.

Our finding that raised cellular l-2HG levels resulted in suppressed expression of amino acid biosynthetic genes led us to consider effects on activating transcription factor 4 (ATF4). ATF4 is a master regulator of amino acid metabolism genes and coordinates the cellular response to nutrient stress. Similar to our findings with amino acid biosynthetic enzymes, L2HGDH restoration led to increased ATF4 protein in multiple RCC lines examined (Figure 2A). Furthermore, this effect was dependent on L2HGDH catalytic activity, as mutant enzyme did not promote ATF4 protein expression (Figure 2B). Consistent with these data, *L2HGDH*-knockout renal epithelial cells demonstrated reduced ATF4 expression (Figure 2C). Given these data, we considered whether l-2HG suppression of amino acid biosynthetic enzymes could, at least in part, be due to suppression of ATF4. In support, we found that expression of ATF4 cDNA in high–l-2HG RCC cells promoted expression of *PHGDH*, an established target gene of ATF4 (Figure 2D). These data prompted us to examine effects on *ATF4* transcript levels. Lowering l-2HG levels in RCC cells via L2HGDH restoration led to increased *ATF4* mRNA levels in 769p and 786-O RCC cells (Figure 2E). As these data supported an epigenetic basis for l-2HG’s effect on amino acid metabolism, we considered α-KG–requiring enzymes implicated in the regulation of amino acid metabolism. The histone lysine demethylase KDM4C promotes mRNA expression of amino acid metabolism genes involved in both biosynthesis and transport (13). KDM4C removes the histone repressive mark H3 lysine 9 trimethylation (H3K9me3) at the *ATF4* mRNA. Furthermore, KDM4C physically interacts with ATF4 protein to promote expression of serine biosynthetic genes including *PHGDH* and *PSAT1*. However, KDM4C’s ability to regulate amino acid metabolism in the setting of an oncometabolite has not been considered. L2HGDH restoration had no effect on KDM4C expression (Supplemental Figure 2A). We attempted a rescue experiment by ablating KDM4C in L2HGDH-restored cells. Using a siRNA pool, KDM4C knockdown resulted in reduced ATF4 with reduced *PHGDH* mRNA (Supplemental Figure 2, B and C). Similarly, KDM4C knockdown with multiple individual siRNA constructs in L2HGDH-restored RCC cells led to reduced ATF4 protein along with reduced expression of serine biosynthetic enzymes PHGDH and PSAT1 (Figure 2, F and G). These rescue data support that l-2HG’s suppression of ATF4/PHGDH/PSAT1 is mediated in part through KDM4C inhibition.

A major mode of regulation of ATF4 is at the level of mRNA translation. The *ATF4* mRNA has multiple upstream open reading frames (uORFs) in the 5′-untranslated region (5′-UTR). Under nutrient-replete conditions, the ATF4 transcript is poorly translated owing to translation of uORFs that are overlapping and out of frame with the ATF4 open reading frame start codon (14, 15). Under stresses such as nutrient starvation, “leaky” scanning by ribosomes allows ATF4 translation within the correct reading frame, which increases ATF4 protein (16). We therefore considered l-2HG’s impact on translation of the *ATF4* mRNA using a luciferase-based reporter construct (17). We observed that WT L2HGDH led to increased ATF4 translation in both 769p and 786-O RCC cells (Figure 2H). In contrast, mutant L2HGDH did not increase ATF4 translation. Zhou et al. recently reported that RNA *N*^6^-methylation of adenosine (m6A) in the *ATF4* mRNA can impede translation of the open reading frame that produces ATF4 protein (18). The m6A mark is reversible. The mark is laid down by an RNA methyltransferase complex (19). In contrast, m6A can be removed by the RNA demethylases ALKBH5 and FTO (20, 21). As both ALKBH and FTO require α-KG as a cofactor for demethylase activity, we considered whether l-2HG could impact the expression of amino acid metabolism factors through RNA methylation.

### l-2HG promotes mRNA m6A methylation.

**Figure 3:**
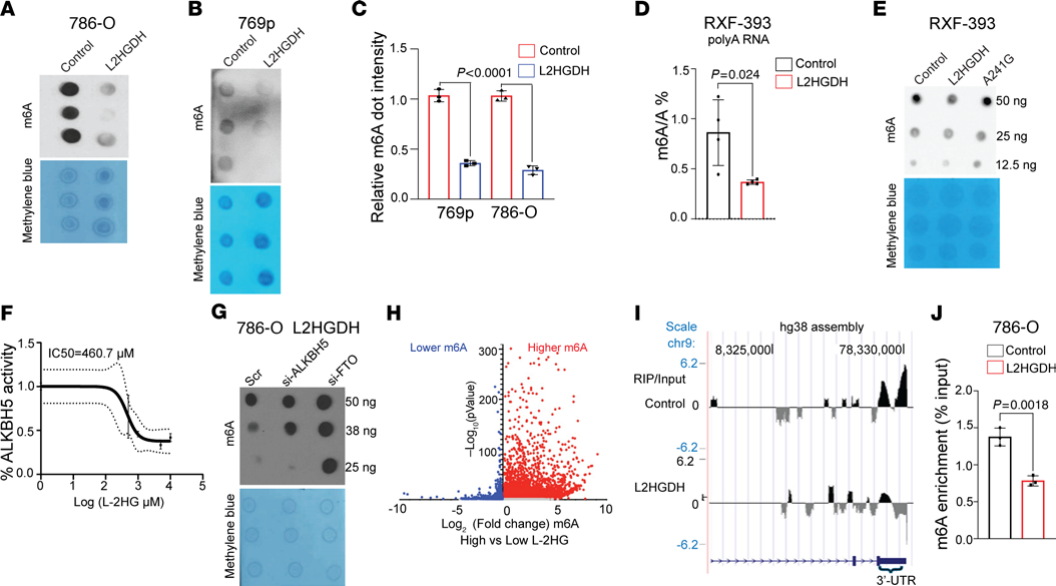
l-2HG promotes mRNA m6A methylation in RCC. (**A** and **B**) m6A dot blot of 100 ng mRNA isolated from 786-O (**A**) and 769p (**B**) cells (*n =* 3, biological replicates) stably expressing control vector or L2HGDH (WT). Methylene blue blot serves as loading control. (**C**) Quantification of m6A normalized to methylene blue as shown in **A** and **B**. Data are shown as mean ± SD from *n =* 3 biological replicates. (**D**) LC-MS/MS analysis of m6A levels in mRNA from RXF-393 cells stably expressing control vector or L2HGDH. Data are presented as ratio of m6A to unmodified adenosine (m6A/A). Data are shown as mean ± SD from *n =* 4 biological replicates. (**E**) m6A dot blot of mRNA isolated from RXF-393 cells stably expressing control vector, L2HGDH WT, or L2HGDH mutant (A241G). (**F**) In vitro ALKBH5 activity with increasing concentration of l-2HG. Each data point represents mean ± error values of *n =* 2 technical replicates. (**G**) 786-O cells stably transduced with L2HGDH cDNA were transiently treated with scramble siRNA (Scr) or siRNAs targeting ALKBH5 and FTO (52 hours). mRNA was harvested and assessed for m6A via dot blot. (**H**) Volcano plot of m6A-Seq demonstrating relative changes in m6A peaks in mRNAs isolated from high–l-2HG control versus low–l-2HG (L2HGDH-transduced) 786-O cells *n =* 1 each. Red denotes higher m6A levels in high l-2HG levels. Blue denotes lower m6A levels in high l-2HG levels. (**I**) m6A-Seq analysis of the *PSAT1* mRNA from 786-O control (high–l-2HG) and L2HGDH (low–l-2HG) cells. For each condition, enrichment is displayed as RNA m6A-immunoprecipitated (RIP) normalized to the corresponding input. (**J**) m6A immunoprecipitation RT-qPCR was used to assess m6A enrichment in the *PSAT1* 3′-UTR from 786-O cells stably transduced with the indicated vector. PSAT1-1F/1R primer pair was used. Data are represented as mean ± SD from *n =* 3 biological replicates.

We therefore first examined the impact of raised l-2HG in RCC on global mRNA m6A levels via dot blot assay using an antibody specific to the m6A mark. Lowering l-2HG levels via L2HGDH restoration led to a significant reduction in mRNA m6A levels in multiple lines tested (Figure 3, A–C). As an orthogonal approach, we measured m6A levels via liquid chromatography–mass spectrometry (LC-MS), which also demonstrated that L2HGDH lowered m6A levels (Figure 3D). Furthermore, this effect depended on L2HGDH’s catalytic activity, as the A241G mutant did not lower m6A levels in RCC cells (Figure 3E). l-2HG has previously been shown to inhibit the enzymatic activity of FTO. We therefore examined l-2HG’s effects on the activity of recombinant ALKBH5 on a methylated RNA substrate. As demonstrated in Figure 3F, l-2HG inhibited ALKBH5 activity in a dose-dependent manner. We next considered whether L2HGDH’s lowering of m6A was mediated through ALKBH5 and/or FTO. As demonstrated in Figure 3G, ablation of either FTO or ALKBH5 increased m6A levels in 786-O cells expressing L2HGDH. Given these data, we performed transcriptome-wide profiling of RNA methylation (m6A-Seq) in high–l-2HG (control vector) RCC cells with low–l-2HG (L2HGDH-restored) cells. As compared with dot blot, this analysis can determine the location and intensity of m6A peaks in mRNA. m6A-Seq of high– (control vector) versus low–l-2HG (L2HGDH-restored) cells demonstrated that l-2HG promoted a marked increase in m6A peaks throughout the transcriptome (Figure 3H and Supplemental Table 2). The predominant location of this increase was with the 3′-UTR of mRNAs, consistent with where m6A was most commonly located within transcripts (Supplemental Figure 3A). We did not find a significant effect of l-2HG on m6A on the ATF4 mRNA (data not shown). However, l-2HG did promote m6A accumulation within the 3′-UTR of the transcript for *PSAT1*, which encodes the enzyme that catalyzes the second reaction of serine biosynthesis (Figure 3I). m6A immunoprecipitation followed by RT-qPCR in multiple RCC lines demonstrated that l-2HG promoted m6A accumulation in the 3′-UTR of *PSAT1* mRNA (Figure 3J and Supplemental Figure 3B).

### l-2HG suppresses PSAT1 expression through mRNA m6A.

**Figure 4:**
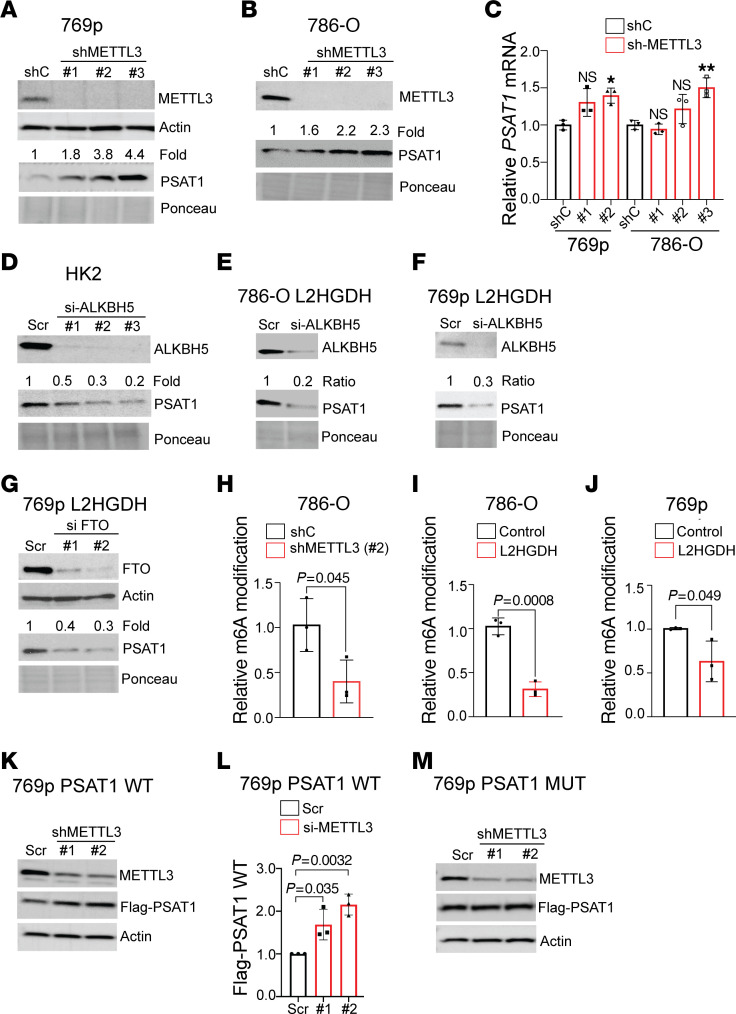
Regulation of PSAT1 expression through l-2HG–induced m6A. (**A** and **B**) Immunoblot of METTL3 and PSAT1 from 769p (**A**) and 786-O (**B**) cells stably expressing the indicated shRNA (shC, control shRNA). Actin and Ponceau serve as loading control. Relative PSAT1 was normalized to Ponceau. (**C**) RT-qPCR analysis of *PSAT1* mRNA expression (normalized to RPLPO) from 786-O and 769p cells stably expressing control shRNA (shC) or METTL3-targeting shRNA. Data are represented as mean ± SD from *n =* 3 biological replicates. *P < 0.05, **P <0.005. (**D**) Immunoblot of ALKBH5 and PSAT1 from HK-2 renal epithelial cells transiently transfected (52 hours) with scramble siRNA (Scr) or siRNAs targeting ALKBH5 (#1, #2, #3). Relative PSAT1 was determined by normalizing to Ponceau loading control. (**E** and **F**) Immunoblot of ALKBH5 and PSAT1 from 786-O/L2HGDH (**E**) and 769p/L2HGDH (**F**) cells transiently transfected (52 hours) with the indicated siRNA Scr, scramble siRNA. Relative PSAT1 was normalized to Ponceau. (**G**) Immunoblot of FTO and PSAT1 from 769p/L2HGDH cells transiently transfected (52 hours) with the indicated siRNA. Relative PSAT1 was normalized to Ponceau. (**H**–**J**) Relative m6A by SELECT at the GGACT site within the *PSAT1* 3′-UTR. (**H**) Relative m6A in 786-O cells transduced with control shRNA shC or shMETTL3. (**I** and **J**) Relative m6A levels in 786-O (**I**) and 769p (**J**) cells transduced with vector control or L2HGDH. Data are shown as mean ± SD from *n =* 3 biological replicates. (**K** and **L**) 769p cells were stably transduced with FLAG-tagged PSAT1 (WT) construct. Cells were transiently transfected (52 hours) with the indicated siRNA followed by immunoblotting (**K**). (**L**) Quantitative densitometry of FLAG levels normalized to actin. Data are represented as mean ± SD from *n =* 3 biological replicates. ANOVA was used, and Tukey’s post hoc *P* values are shown. (**M**) 769p cells were stably transduced with FLAG-tagged PSAT1 (MUT) construct. Cells were transiently transfected (52 hours) with the indicated siRNA followed by immunoblotting.

We next sought to determine the role of l-2HG–induced m6A in regulating PSAT1 expression. We therefore knocked down METTL3, which is the catalytic subunit of the m6A methyltransferase complex. METTL3 knockdown led to increased PSAT1 protein expression (Figure 4, A and B). As expected, METTL3 knockdown led to reduced mRNA m6A levels (Supplemental Figure 3, A and B). Although METTL3 knockdown trended toward a slight increase in *PSAT1* mRNA levels, these data did not reach statistical significance (Figure 4C). Given these data, we next determined whether RNA m6A demethylases can regulate PSAT1 expression. In HK-2 renal epithelial cells, which have low basal l-2HG levels, knockdown of the m6A demethylase ALKBH5 led to reduced PSAT1 (Figure 4D). L2HGDH restoration in high–l-2HG cells increased PSAT1 levels. We determined whether this effect could be rescued by m6A demethylase ablation via either ALKBH5 or FTO knockdown. In both 786-O L2HGDH and 769p L2HGDH cells, ALKBH5 knockdown led to reduced PSAT1 (similar to that in high–l-2HG cells) (Figure 4, E and F). Furthermore, FTO knockdown also reduced PSAT1 in 769p L2HGDH cells (Figure 4G). m6A methylation occurs on DRACH motifs (D = G/A/U; R = G/A; H = A/U/C; central A is methylated). The most common sequence for this motif is GGACT. Notably, the 3′-UTR of *PSAT1* has a single GGACT site (Supplemental Figure 4C). Single-nucleotide-resolution mapping of m6A in HEK293 cells by Linder et al. using both cross-link–induced mutational sites and cross-link–induced truncation sites identified m6A at this site (Supplemental Figure 4D) (22). As m6A immunoprecipitation has a resolution of 100–200 bp, we used SELECT for single-nucleotide resolution of m6A levels (23). SELECT is based on the premise that m6A (as opposed to unmodified adenosine) hinders both *Bst1* DNA polymerase elongation and *SplintR* nick ligase activity. Oligonucleotides are designed that are immediately upstream and downstream of the RNA m6A site. The oligonucleotides have PCR adaptors, which allows for quantification with qPCR. Sites with higher m6A levels are amplified less efficiently. METTL3 knockdown in high–l-2HG RCC cells led to reduced m6A levels at the GG**A**CT site (bold indicates nucleotide undergoing m6A methylation) within the *PSAT1* 3′-UTR (Figure 4H). We then determined the impact of L2HGDH restoration on m6A levels at this site. In multiple RCC lines, L2HGDH restoration (i.e., lowering l-2HG levels) resulted in significantly reduced m6A levels at this site (Figure 4, I and J). We next determined the significance of this methylation with regard to PSAT1 expression. We generated a *PSAT1* cDNA construct that contained the 3′-UTR and stably expressed this construct in high–l-2HG RCC cells. Constructs included an N-terminus FLAG tag to differentiate from endogenous PSAT1. Similar to endogenous PSAT1, knockdown of m6A methyltransferase subunit METTL3 led to increased expression of tagged PSAT1 (Figure 4, K and L). In tandem, we expressed a *PSAT1* mutant in which the GG**A**CT site in the 3′-UTR is mutated to GG**T**CT (bold indicates A to T point mutant). In contrast to WT, mutant PSAT1 levels did not change with METTL3 ablation (Figure 4M). Collectively, these data demonstrate that l-2HG promotes m6A methylation in the 3′-UTR of *PSAT1* mRNA, which results in lower expression of this serine biosynthetic enzyme.

### l-2HG promotes a serine liability in RCC.

**Figure 5:**
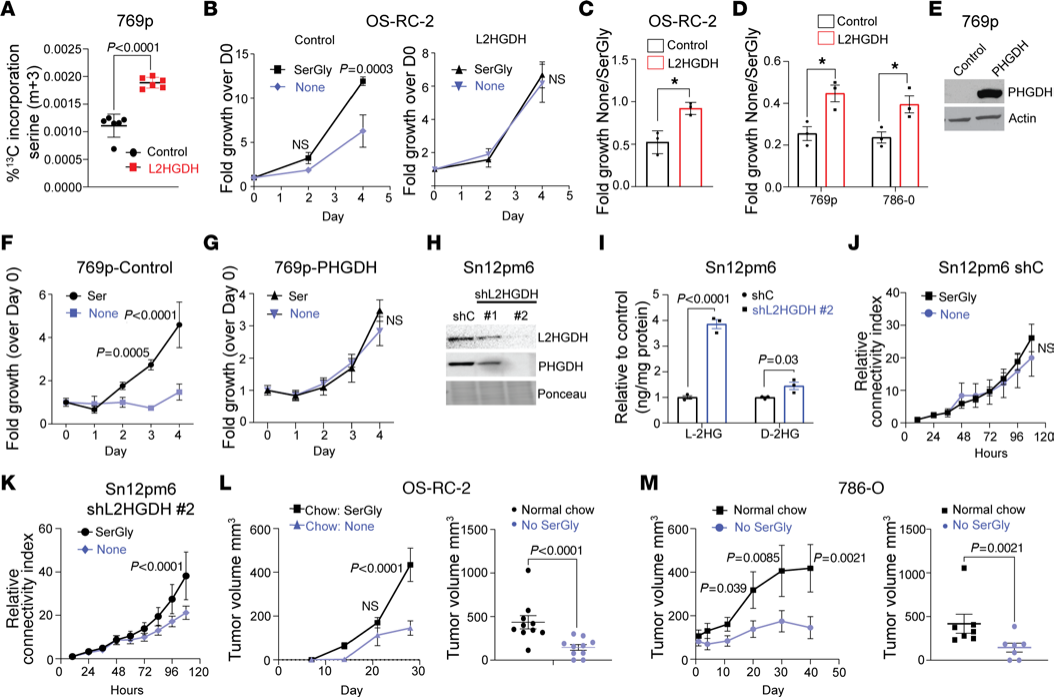
High l-2HG causes exogenous serine dependency in RCC cell lines. (**A**) LC-MS analysis of serine synthesis (m+3) from labeled glucose (U-^13^C). *n =* 6 biological replicates. (**B**) Proliferation of OS-RC-2 cells stably transduced with the indicated vector (left, control; right, L2HGDH) in media with or without SerGly. *n =* 3 biological replicates. Repeated-measures analysis was conducted, and *P* values reflect Tukey’s post hoc comparisons. (**C** and **D**) Relative growth ratio (–SerGly/+SerGly) at day 4 in RCC cells transduced with the indicated vector. *n =* 3 biological replicates. *P < 0.05. (**E**) Immunoblot of PHGDH from 769p cells stably expressing control vector or PHGDH cDNA. (**F** and **G**) Proliferation of 769p cells transduced with control vector (**F**) or PHGDH (**G**) in media with or without SerGly. *n =* 3 biological replicates of each group. Repeated-measures analysis was conducted, and *P* values reflect Tukey’s post hoc comparisons. (**H**) Immunoblot of Sn12pm6 cells stably expressing the indicated shRNA. (**I**) Relative l/d-2HG levels in Sn12pm6 cells transduced with the indicated shRNAs (shC, control). *n =* 3 biological replicates. (**J** and **K**) Cell proliferation (by connectivity index) was measured from Sn12pm6-Scr (**J**) and Sn12pm6-shL2HGDH (**K**) cells grown with or without SerGly. *n =* 4 biological replicates of each group. Repeated-measures analysis was conducted, and *P* values reflect Tukey’s post hoc comparisons. (**L**) Left: After subcutaneous implantation of OS-RC-2 cells, mice were randomly distributed and fed chow with or without SerGly (*n =* 10 per group) for 4 weeks. Right: Tumor volume at day 28. Repeated-measures analysis was performed followed by Tukey’s post hoc comparisons at each time point. (**M**) 786-O xenografts were established in flanks of nude mice. Left: After the average tumor size reached 100 mm^3^, mice were fed chow with or without SerGly (*n =* 7 per group) and followed over time (left). Right: Xenograft size (day 40). Repeated-measures analysis was performed followed by Tukey’s post hoc comparisons at each time point Data are shown as (**A**–**D**, **F**, **G**, **J**, and **K**) mean ± SD and (**I**, **L**, and **M**) mean ± SEM.

Given that l-2HG suppressed the expression of serine biosynthetic enzymes, we next assessed the sequelae of this finding. The starting substrate for de novo serine biosynthesis is the glycolytic intermediate 3-phosphoglycerate. We therefore used fully labeled ^13^C-glucose (U-^13^C) with LC-MS to assess serine biosynthesis as a function of l-2HG. L2HGDH restoration in RCC cells resulted in enhanced de novo serine biosynthesis as determined by increased production of fully labeled (m+3) serine (Figure 5A and Supplemental Table 3). In addition, we found that L2HGDH led to increased levels of the m+2 isotopologue of serine (Supplemental Figure 5A), which can be generated via the interconversion of serine and glycine via serine hydroxymethyltransferases (SHMTs). As these data indicate that serine biosynthesis is reduced in the setting of raised l-2HG, we next considered the effects of serine availability on the growth of RCC cells as a function of l-2HG status. We therefore assessed proliferation in the presence and absence of serine and glycine (SerGly). Glycine is also modulated as it can be converted to serine by SHMTs as noted above. The proliferation of control OS-RC-2 cells (high l-2HG) was significantly reduced upon depletion of SerGly from the media (Figure 5B). These effects were sustained in multiple high–l-2HG RCC lines cultured for a longer period (Supplemental Figure 5B). Consistent with prior studies, L2HGDH restoration led to reduced proliferation. However, the proliferation of L2HGDH-restored OS-RC-2 cells was not impacted by SerGly depletion from the media (Figure 5, B and C). Similarly, in other RCC lines tested, the effect of SerGly deprivation on proliferation was significantly greater in control (high–l-2HG) cells as compared with L2HGDH-restored (low–l-2HG) cells (Figure 5D and Supplemental Figure 5, B and C). Expression of *PHGDH* cDNA in high–l-2HG RCC cells could rescue the ability of cells to proliferate in the absence of serine in the media (Figure 5, E–G). We next performed loss-of-function studies in Sn12pm6 RCC cells, which have retained L2HGDH expression. Knockdown of L2HGDH expression in these cells led to reduced expression of PHGDH (Figure 5H) and the expected increase in cellular l-2HG levels (Figure 5I). The proliferation of control vector–transduced cells was not impacted by SerGly deprivation (Figure 5J). In contrast, the proliferation of L2HGDH-knockdown cells was reduced with SerGly deprivation (Figure 5K). Correspondingly, knockdown of PHGDH in Sn12pm6 cells rendered proliferation sensitive to SerGly deprivation (Supplemental Figure 5, D and E). In agreement, the proliferation of L2HGDH-restored RCC cells in media lacking SerGly was reduced with PSAT1 knockdown (Supplemental Figure 5, G and H).

Given these in vitro data, we next considered the effects of limiting serine availability to the growth of high–l-2HG RCC in vivo given the reliance of tumor cells on exogenous serine to maintain proliferation. A potential source of serine is through the diet. We therefore considered the effects of SerGly depletion from the diet on the growth of high–l-2HG RCC xenografts. Mice were stratified to receive chow with or without SerGly at the time of subcutaneous injection of OS-RC-2 cells. In mice fed chow without SerGly, tumor xenograft growth was delayed (Figure 5L, left). Furthermore, end-of-study xenograft tumor size was significantly lower in mice fed chow lacking SerGly (Figure 5L, right). As a complementary approach, we initially injected 786-O cells in mice fed normal chow and allowed xenografts to establish. Mice were then stratified to receive chow with or without SerGly. Further xenograft growth was largely blocked in mice that were switched to a diet lacking SerGly (Figure 5M, left), with significantly smaller xenograft size at end of study (Figure 5M, right).

### Exogenous serine is required for glutathione synthesis in high–l-2HG RCC.

**Figure 6:**
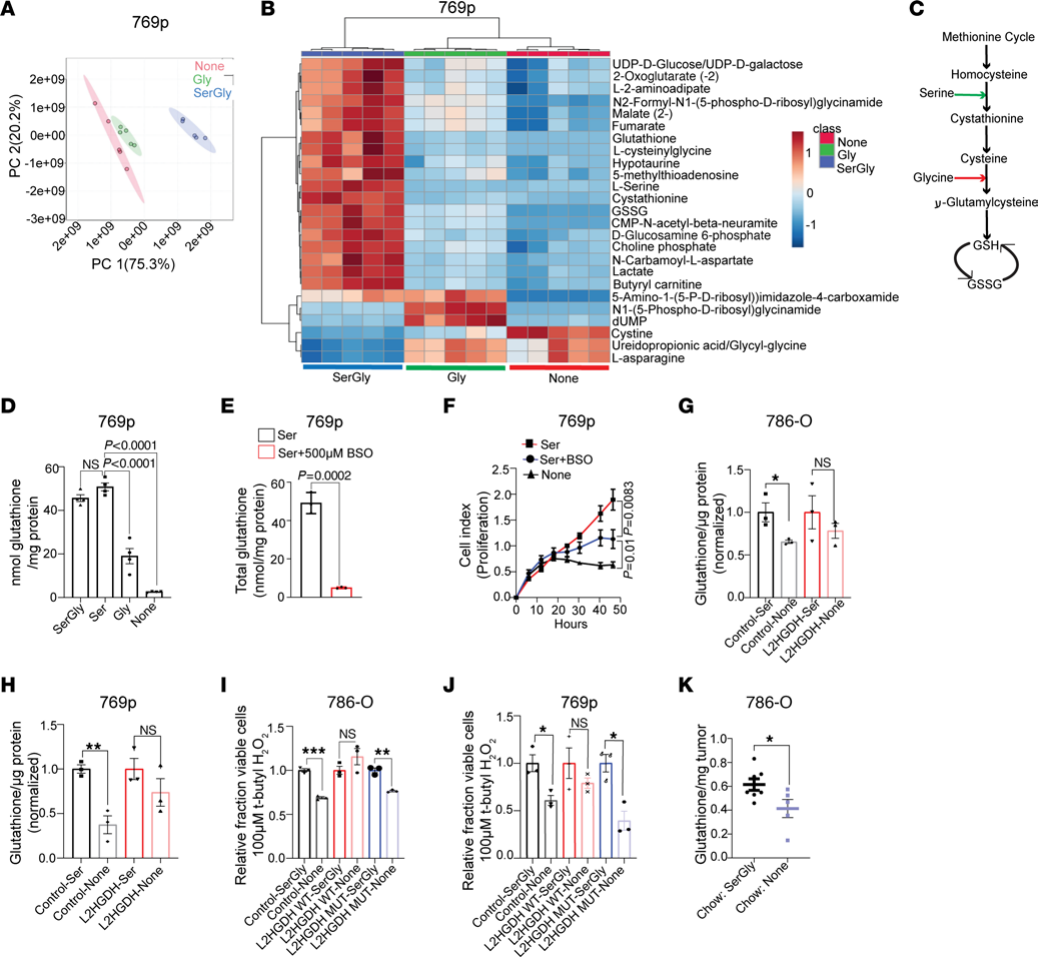
Exogenous serine is required for glutathione synthesis in RCC. (**A**) Principal component plot from partial least-squares discriminant analysis of metabolites from 769p cells grown in media containing both serine and glycine, glycine only, or no serine and glycine (None) (*n =* 5 biological replicates per group). (**B**) LC-MS analysis of metabolites extracted from the 3 groups of 769p cells described in **A**. (**C**) Schematic view of serine and glycine incorporation into glutathione (GSH). (**D**) Analysis of cellular GSH levels from 769p cells cultured for 24 hours in media as indicated. Results are presented as mean ± SEM from *n =* 4 biological replicates of each group. ANOVA *P* value < 0.0001. Post hoc Tukey’s *P* values are shown. (**E**) Analysis of cellular GSH levels from 769p cells cultured for 24 hours in serine-containing media with or without BSO. Data are presented as mean ± SD from *n =* 3 biological replicates per group. (**F**) Cell proliferation as determined by connectivity index in 769p cells grown as indicated. Each data point represents mean ± SD from *n =* 4 biological replicates of each group. (**G** and **H**) GSH levels of 786-O (**G**) and 769p (**H**) cells stably expressing control vector or L2HGDH and cultured for 24 hours in media with or without (None) serine. Data are shown as mean ± SEM from *n =* 3 biological replicates in each group. *P < 0.05, **P < 0.005. (**I** and **J**) Relative fraction of viable cells in 786-O (**I**) and 769p (**J**) cells stably expressing the indicated vector and treated with 100 mM *tert*-butyl H_2_O_2_ for 24 hours in the presence or absence of SerGly. Results are presented as mean ± SEM from *n =* 3 biological replicates of each group. *P < 0.05, **P < 0.005, ***P < 0.0001. (**K**) Quantification of GSH content in 786-O xenografts in mice fed chow with or without SerGly. Xenografts are from data presented in Figure 5M. Results are presented as mean ± SEM. *P < 0.05.

Based on these data, we considered serine’s contribution to tumor biology using steady-state metabolomics by comparing high–l-2HG RCC cells cultured with media under the following conditions: (a) with SerGly, (b) without SerGly, and (c) with glycine alone. As demonstrated by principal component analysis, the metabolomes of cells cultured with SerGly were distinct from those of cells with the other culture conditions (Figure 6A). High–l-2HG 769p RCC cells cultured in the presence of SerGly had significantly higher steady-state levels of glutathione (GSH) as well as metabolites derived from GSH, including GSH disulfide (GSSG) and l-cysteinylglycine (Figure 6B and Supplemental Table 4). These data are particularly relevant as GSH is among the most enriched metabolites in RCC as compared with normal kidney (over 100-fold) in multiple metabolomic data sets (24, 25). GSH is a tripeptide consisting of the amino acids glutamate, cysteine, and glycine. Serine can directly contribute to GSH through its conversion to glycine via SHMTs. GSH synthase condenses glycine and γ-glutamylcysteine to form GSH (Figure 6C, red arrow). Alternatively, serine can directly contribute to cysteine pools via transsulfuration, which can then be incorporated into GSH (Figure 6C, green arrow). Exogenous serine alone was able to maintain GSH pools similar to the levels of cells cultured with both serine and glycine in the media (Figure 6D). In contrast, RCC cells cultured with glycine but without serine were not able to maintain cellular GSH pools (Figure 6D). Cells cultured without either serine or glycine had even lower levels of GSH. Using ^13^C_3_ serine in concert with liquid chromatography–tandem mass spectrometry (LC-MS/MS) analysis, we examined whether serine contributes directly to the GSH tripeptide. Label from serine contributed primarily to m+1 and m+2 pools of GSH (Supplemental Figure 6C). Isotopic pattern analysis of the m+1 species demonstrated that serine contributed to the glycine moiety and, to a lesser extent, the cysteine moiety (Supplemental Figure 6D). Similar results were found upon analysis of the m+2 species (Supplemental Figure 6E). These data are particularly relevant to tumor cells including RCC given that redox homeostasis is critical to maintaining proliferation. As exogenous serine is required to support proliferation of high–l-2HG RCC cells, we considered whether this was mediated through serine’s support of GSH pools and redox homeostasis. We therefore examined the effects of buthionine sulfoximine (BSO), which blocks GSH synthesis via inhibition of γ-glutamylcysteine synthetase. BSO treatment led to a significant reduction in cellular GSH pools despite the presence of serine in the media (Figure 6E). Furthermore, exogenous serine’s promotion of RCC proliferation was diminished with BSO treatment (Figure 6F). Serine may also contribute to proliferation via purine biosynthesis through provision of one-carbon units and glycine. We therefore examined whether exogenous formate (to replenish one-carbon metabolism) and/or glycine could support proliferation in the absence of exogenous serine. In multiple high–l-2HG RCC lines examined, formate plus glycine treatment could not rescue proliferation in the absence of serine (Supplemental Figure 6C).

Given these data, we next examined the impact of l-2HG on GSH pools as a function of nutrient availability. Similarly to parental cells, control vector (high–l-2HG) cells demonstrated a significant reduction of GSH levels when cultured in the absence of serine (Figure 6, G and H, compare lanes 1 and 2). In contrast, L2HGDH-restored (low–l-2HG) cells were able to maintain GSH pools in the absence of exogenous serine (Figure 6, G and H, compare lanes 3 and 4). We next examined the impact of l-2HG levels on the ability to withstand oxidative stress as a function of SerGly availability. High–l-2HG (control) 786-O RCC cells were sensitive (i.e., reduced viability) to the ROS inducer *tert*-butyl H_2_O_2_ upon SerGly deprivation (Figure 6I, compare lanes 1 and 2). Similar results were found in high–l-2HG RCC cells expressing mutant L2HGDH (Figure 6I, compare lanes 5 and 6). In contrast, SerGly deprivation did not sensitize WT L2HGDH-restored cells (low l-2HG) to *tert*-butyl H_2_O_2_ (Figure 6I, compare lanes 3 and 4). Similar results were found in 769p cells (Figure 6J). Next, we determined the relevance of dietary SerGly to GSH pools in vivo. 786-O xenografts in mice fed chow lacking SerGly had lower total GSH levels as compared with xenografts from mice fed chow replete with SerGly (Figure 6K).

### Translational relevance of raised l-2HG to serine metabolism.

**Figure 7:**
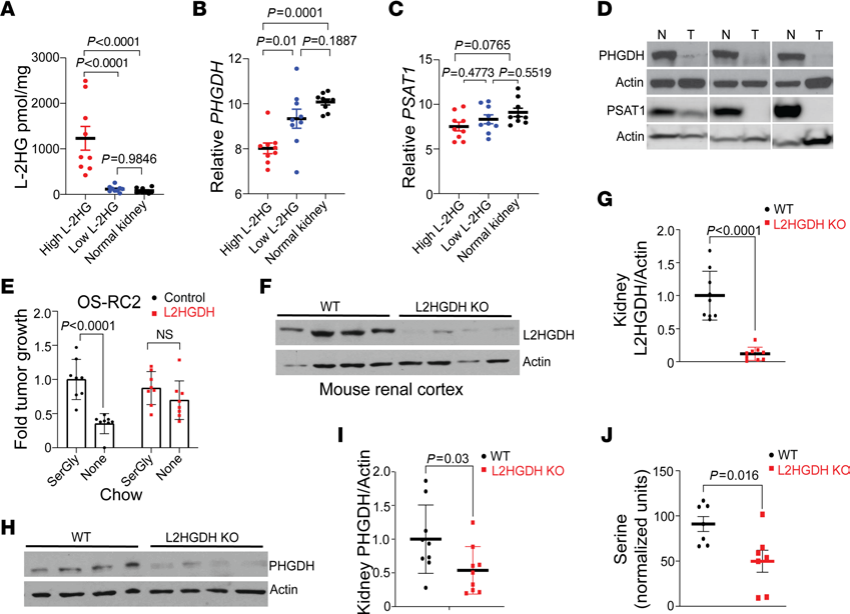
Translational relevance of raised l-2HG in RCC. (**A**) LC-MS analysis of l-2HG content (normalized to total protein content) from patient tissues harvested from normal kidneys (black dots), high–l-2HG tumors (red dots), or low–l-2HG tumors (blue dots). Data are presented as mean ± SEM from *n =* 9 samples. Data were analyzed by 1-way ANOVA followed by post hoc Tukey’s honestly significant difference test. ANOVA *P* value < 0.0001 (*F* = 18.54). Post hoc analysis *P* values are shown. (**B** and **C**) mRNA expression of *PHGDH* (**B**) and *PSAT1* (**C**) from the samples in **A**. Data are presented as mean ± SEM. Data were analyzed by 1 way ANOVA followed by post hoc Tukey’s honestly significant difference test. ANOVA *P* values for **B** and **C** are 0.0002 (*F* = 12.91) and 0.0932 (*F* = 2.624), respectively. Post hoc analysis *P* values are shown. (**D**) Immunoblot analysis of PHGDH and PSAT1 from patient-matched (*n =* 3) high–l-2HG tumors and adjacent normal kidney. The Western blot in the third PHGDH panel is the same blot with shorter exposure time. (**E**) Relative tumor size of OS-RC-2 xenografts (with or without L2HGDH) at 4 weeks in mice fed chow with or without SerGly. After average tumor size reached 100 mm^3^, mice were randomly distributed into 2 groups (*n =* 8 per group). Data are shown as mean ± SEM. (**F** and **G**) Immunoblot of L2HGDH protein (**F**) and densitometric quantification (**G**) in renal cortical tissue from WT and global-L2HGDH-KO mice. Data were normalized to actin. Data in **G** are presented as mean ± SEM. (**H** and **I**) PHGDH immunoblot and quantification from WT and L2HGDH-KO mouse renal cortical tissues. Data were normalized to actin. Data in **I** are presented as mean ± SEM. (**J**) Gas chromatography-MS analysis of serine from WT and global-L2HGDH-KO renal cortical tissues. Data are presented as mean ± SEM from *n =* 7 mice in each group.

These data prompted us to determine the translational relevance of our findings. We first analyzed the expression of serine biosynthetic genes in high–l-2HG RCC, low–l-2HG RCC, and normal kidney. l-2HG levels of samples are provided in Figure 7A. Consistent with prior studies, normal kidney tissues have low l-2HG levels (5, 12, 26). High–l-2HG RCC demonstrated reduced *PHGDH* mRNA expression as compared with both low–l-2HG RCC and normal kidney (Figure 7B). Furthermore, high–l-2HG RCC had lower expression of *PSAT1* mRNA than normal kidney (Figure 7C). Although low–l-2HG RCC tumors trended toward lower *PSAT1* mRNA as compared with normal kidney, these data did not reach statistical significance. In agreement with these data, both PHGDH and PSAT1 protein expression was lower in high–l-2HG RCC as compared with patient-matched adjacent uninvolved normal kidney (Figure 7D). Given our in vitro data demonstrating the importance of exogenous serine to the proliferation of high–l-2HG RCC cells, we determined the relevance of this finding in vivo in mice fed chow with or without SerGly. As with parental cells, the size of control vector (high–l-2HG) OS-RC-2 xenografts was sensitive to dietary SerGly deprivation (Figure 7E, compare lanes 1 and 2; and Supplemental Figure 7). However, the size of L2HGDH-restored xenografts was not affected by dietary SerGly deprivation (Figure 7E, compare lanes 3 and 4; and Supplemental Figure 7). We next examined renal tissues from mice with whole-body *L2hgdh* knockout, which we recently reported demonstrated elevated l-2HG levels and reduced L2HGDH protein (Figure 7, F and G) (27). Kidneys from knockout mice demonstrated lower PHGDH protein expression (Figure 7, H and I). We next determined serine levels from knockout and WT renal tissues. To exclude potential contribution from the diet, mice were fasted before renal harvest. As demonstrated in Figure 7J, serine levels were significantly lower in *L2hgdh*-knockout kidneys as compared with kidneys from littermate WT mice.

## Discussion

We find that raised l-2HG levels result in a dependence on exogenous serine for renal tumor proliferation, in vivo growth, and redox homeostasis. To the best of our knowledge, this is the first report of a metabolic liability in the setting of high l-2HG. With the recognition that tumor cells have increased demands for nutrients including amino acids to support proliferation and biomass accumulation (28), there has been intense interest in identifying metabolic vulnerabilities that can be exploited. There has been particular focus on non-essential amino acids (NEAAs) as tumor cells often exhibit high demands for these nutrients that may outweigh their biosynthetic capacity. This has led to preclinical strategies including modified diets, which serve as proof of principle that restricting an NEAA of interest may have therapeutic potential (29).

While dietary restriction of serine can suppress tumor growth in some preclinical models, others have proven resistant. Using isogenic cell lines, Maddocks et al. demonstrated that *p53* loss can sensitize colon cancer cells to dietary restriction of serine/glycine (30). Apart from this study, the underlying mechanisms that contribute to sensitivity (or resistance) to strategies that deplete serine are poorly understood. Accordingly, the tumor types most likely to benefit from such strategies remain largely unknown. A recent study demonstrated that human pancreatic ductal adenocarcinomas (PDACs), most of which harbor *KRAS* mutations, show loss of PHGDH expression and are therefore sensitive to deprivation of exogenous serine (31). However, the mechanisms that drive this loss remain unclear. In addition, the metabolic sequelae of serine deprivation for tumor cells are highly context dependent. For example, serine deprivation in low-PHGDH PDAC tumor cells does not demonstrate appreciable effects on GSH levels (31). In contrast, our studies reveal that exogenous serine is critical to maintain GSH pools in RCC cells. Our findings are particularly relevant in light of multiple metabolomic data sets demonstrating that GSH is among the most enriched metabolites in kidney cancer as compared with normal kidney (24, 25, 32). Notably, glycine alone cannot maintain GSH pools in RCC cells. Our findings reveal that a major contribution of serine to GSH pools in RCC is through transsulfuration. The rate-limiting step of this pathway generates cystathionine. In turn, cystathionine is converted to cysteine, which can then be incorporated into the GSH tripeptide. Cysteine can also be generated from cystine, which can be taken up by the xCT transporter system (33). However, the media conditions used throughout this study contained cystine, indicating that the xCT system is not sufficient to generate the cysteine required for GSH synthesis.

Our data reveal a dual mechanism by which serine biosynthetic enzymes are suppressed in RCC via remodeling of both the epigenome and the epitranscriptome through the combined inhibition of the histone demethylase KDM4C and the RNA m6A demethylases ALKBH5 and FTO. KDM4C is known to require α-KG as a cofactor to promote amino acid biosynthetic/transport genes (13). However, the impact of an oncometabolite on the KDM4C-driven amino acid metabolism gene expression program has not been considered. m6A has previously been implicated in regulating ATF4. Zhou et al. proposed that ALKBH5 demethylation of an m6A site in the 5′ region of the murine *Atf4* mRNA promotes translation (18). However, this m6A site is not conserved in humans, suggesting the possibility that m6A regulation of ATF4 may be species-specific (34). Our data reveal a role for RNA methylation in regulating the serine biosynthetic enzyme gene *PSAT1*. Despite recent interest in m6A, most of the profiling studies to date do not provide single-nucleotide resolution, as they primarily depend on antibody-based pull-down approaches. Accordingly, the biological significance of most m6A sites remains largely uncharacterized. Our data functionally validate the relevance of an m6A site in the direct regulation of an amino acid biosynthetic enzyme. Our data indicate that *PSAT1* m6A has post-transcriptional effects. Although m6A has been implicated in mRNA translation, the underlying mechanisms are poorly understood and warrant further study. Our studies reveal that l-2HG’s effects converge on serine biosynthetic enzyme expression through multiple regulatory levels (i.e., both histone and RNA methylation). Our data are, to the best of our knowledge, the first to demonstrate loss of expression of multiple serine biosynthetic enzymes in a human cancer.

Our findings present two key questions. First, how do high–l-2HG RCCs meet their serine requirements? Our data reveal that diet is one potential source of serine for RCC tumors. An alternate consideration is endogenous sources of serine. The most relevant would be the kidney. A recent study analyzing metabolite exchange in pigs demonstrated that the kidney is a major contributor to systemic serine pools (35). These data are consistent with studies on isolated renal tubules from rats (36). Prior studies indicate that serine may be generated from the de novo synthetic pathway that utilizes the glycolytic intermediate 3-phosphoglycerate as the starting substrate. An alternate pathway implicated in the kidney is the glycine cleavage system, which can generate serine when coupled with serine hydroxymethyltransferases (SHMTs). Targeting these endogenous sources of systemic serine could represent a strategy to target tumors with suppressed serine biosynthetic enzyme expression as compared with strategies to deplete serine from the diet, which may be more difficult to achieve in the clinic. The second question our findings raise is whether there is a benefit to loss of serine biosynthetic enzymes in RCC tumor cells? In addition to generating serine, the pathway generates other metabolites, including α-KG. In fact, this pathway can be a major contributor to α-KG pools in embryonic stem cells (37). In the setting of RCC, α-KG generated during de novo serine biosynthesis would be expected to antagonize l-2HG’s effects. Therefore, one theoretical advantage of suppressing this pathway is to maintain a higher ratio of l-2HG to α-KG. Future studies to investigate the potential advantages, if any, of suppressing this pathway in the context of l-2HG and other oncometabolites are warranted.

We recognize limitations of our study. Although our study focused on serine, there may be alternate liabilities that ensue in the setting of high l-2HG. We also recognize that there may be additional mechanisms that cooperate with l-2HG to suppress serine biosynthetic enzyme expression in kidney cancer. RCC is known for low mutation burden. Hence, alternate epigenetic and/or epitranscriptomic mechanisms likely contribute to suppression of serine biosynthetic enzymes in RCC.

In summary, our studies reveal a metabolic liability that results from the dual activities of l-2HG on the epigenome and epitranscriptome. The combined analysis of our models, supported by our findings in patient tissues, demonstrates a dependence on exogenous serine in the setting of raised l-2HG. Our biochemical studies demonstrate that this renders high–l-2HG kidney cancer cells dependent on exogenous serine to maintain cellular pools of GSH, a key metabolite required for proliferation and redox homeostasis. Our findings provide novel mechanistic insight into metabolic rewiring induced by this metabolite. Accordingly, they provide rationale to pursue strategies that limit serine availability for l-2HG–driven kidney tumors.

## Methods

### Sex as a biological variable.

Our study examined male and female animals, and similar findings are reported for both sexes.

### Cell culture.

Renal cell lines were acquired from ATCC or the National Cancer Institute. The OS-RC-2 cell line was acquired from RIKEN BioResource Research Center Cell Bank (https://cell.brc.riken.jp/en/). All cells were screened for mycoplasma after their purchase. Cells were passaged for less than 3 months after resuscitation. All media were completed by addition of 10% FBS (Atlanta Biologicals S11550) and 1× penicillin-streptomycin supplements (Thermo Fisher Scientific MT30002CI). HEK293T cells were maintained in complete DMEM (4.5 g glucose per L) (Thermo Fisher Scientific MT-10-017-CV), and all other cells were maintained in complete RPMI 1640 (Thermo Fisher Scientific MT15040CM). For the studies that required serine/glycine deprivation, RPMI 1640 (Thermo Fisher Scientific 50-190-8105) was supplemented with 10% dialyzed FBS (Thermo Fisher Scientific SH3007903), 1× penicillin-streptomycin, 30 mg/L l-serine, 10 mg/L glycine, 2 g/L glucose (MilliporeSigma G7021), and 1× vitamin solution (Thermo Fisher Scientific 11120052).

### Mice.

C57BL/6 mice with CRISPR/Cas9–mediated global knockout of *L2hgdh* that we reported previously (Brinkley et al., ref. 27) were used in this study. Both male and female mice were used in this study. Mice were bred and maintained under specific pathogen–free conditions at the University of Alabama at Birmingham following approved protocols. For in vivo xenograft studies, BALB/c nude mice (strain code 194) were purchased from Charles River Laboratories. All animals had ad libitum access to food and water and were maintained at 22°C–24°C on a 12-hour light/12-hour dark cycle. For in vivo SerGly restriction experiments, animals were fed irradiated control SerGly-free chow obtained from Lab Animal Supplies Inc. Animals were observed daily for health status, and any mice that met IACUC criteria for euthanasia were immediately euthanized.

### Chemicals and reagents.

The following chemicals and reagents were used in this study: d-glucose U-^13^C_6_, 99% (Cambridge Isotope Lab CLM-1396-0.5); *tert*-butyl hydroperoxide (MilliporeSigma 458139-100ML); Trizol (Life Technology 15596-018); RNA Miniprep (Zymo Research R2052); High-Capacity cDNA Synthesis Kit (Thermo Fisher Scientific 43-688-14); TaqMan gene expression master mix (Thermo Fisher Scientific 43-690-16); SYBR Green master mix (Thermo Fisher Scientific 4367659); Blot Stain Blue (MilliporeSigma B-1177); 20× RNase free SSC (Thermo Scientific AM9763); nitrocellulose membrane (Bio-Rad 1620112); PVDF membrane Immobilon-FL (Millipore IPFL00010).

### Plasmids and lentivirus.

Plasmid DNAs for lentivirus production were transfected to HEK293T cells as described earlier (38). Supernatants from transfected HEK293T cells were used to transduce target cells. Control vector, L2HGDH (wild type), and L2HGDH-mutant (mutation at A241G) constructs were described previously (12). ATF4 (BC022088), PHGDH (BC000303), and PSAT1 (BC018129) cDNAs were purchased from transOMIC and placed into LV2606 backbone. Control (SHC016-1EA) and gene-targeted mission shRNAs in pLKO.1-puro backbone were purchased from MilliporeSigma: shMETTL3 (TRCN0000034717, TRCN0000289812, TRCN0000289814); shL2HGDH (TRCN0000064325, TRCN0000064326); shPHGDH (TRCN0000028501, TRCN0000233032). For CRISPR/Cas9 knockout, lentiCRISPR v2 (Addgene 52961) plasmid was linearized by BsmBI v2 (New England Biolabs [NEB] R0739S), dephosphorylated, and gel-purified. Next, annealed oligonucleotides for L2HGDH-targeted (gRNA-3F, -3R) or control guide RNAs (gRNAs) (Control_gRNA_F/R) were phosphorylated, ligated to the linearized plasmid, and propagated in *E*. *coli*. Control and targeted gRNAs used in this study are listed in Supplemental Table 5. Plasmids were isolated and sequence-verified before use. Lentivirus-transduced cells were selected with puromycin.

### Gas chromatography–MS analysis of cellular l-2HG.

Cells were briefly washed in ice-cold PBS, followed by rapid freezing in liquid nitrogen. Samples were added to pre-tared 2 mL screw-cap tubes containing 1.4 mm ceramic beads and massed, and 800 μL of −20°C methanol with 2 μg/mL of both d4-succinic acid and disodium (*R*,*S*)-[2,3,3-^2^H_3_]-2-hydroxyglutarate ([^2^H_3_]-2HG) (C/D/N Isotopes) was added to the tubes. Samples were homogenized and incubated at −20°C for 1 hour. Samples were then centrifuged to remove insoluble debris, and the supernatant dried in a vacuum centrifuge. The extract was then derivatized using a previously described method to quantify d-2HG and l-2HG levels (39). Samples were injected into an Agilent 7890B/7250 GC/Q-TOF instrument (1:10 split ratio) equipped with a Phenomenex ZB5-5 MSi column using a Gerstel MPS autosampler by previously described methods (40). Data were analyzed using MassHunter Qualitative Analysis and MassHunter Quantitative Analysis.

### Mouse tissue gas chromatography–MS.

Mice were fasted for 6 hours before sacrifice and tissue isolation. Tissues were briefly washed in ice-cold DPBS followed by rapid freezing in liquid nitrogen. Gas chromatography–MS analysis was performed as previously described (40).

### Tissue 2HG enantiomer analysis (d- and l-2HG quantification) by LC-MS/MS.

Samples were analyzed as previously described (41). Briefly, enantiomer analyses were performed following derivatization with diacetyl-l-tartaric acid followed by liquid chromatography–tandem mass spectrometry (LC-MS/MS) analysis and normalized to protein levels.

### LC-MS preparation and method.

Two million cells per 10 cm culture plate were grown for 24 hours in 7 mL RPMI 1640 (Teknova) supplemented with 10% dialyzed FBS (HyClone), 30 mg/L serine, 10 mg/L glycine, and 5.5 mM d-glucose. Cells were then thoroughly washed with DPBS and grown for 24 hours in 7 mL RPMI 1640 (Teknova) supplemented with 10% dialyzed FBS, 30 mg/L serine, 10 mg/L glycine, and 5.5 mM (U-^13^C_6_) d-glucose. Cells were then washed with ice-cold 0.9% NaCl in molecular-grade water and lysed in ice-cold 80% (vol/vol) LC-MS–grade methanol (Thermo Fisher Scientific). Lysed cells were scraped into 1.5 mL microcentrifuge tubes and stored at –80°C. The cell lysate was spun at full speed (16,000*g*) for 20 minutes at 4°C. Supernatant was isolated and dry-vacuumed at room temperature until no liquid remained. The dry pellet was reconstituted into 30 mL solvent (water/methanol/acetonitrile 2:1:1, vol/vol/vol), and 3 μL was further analyzed by LC-MS.

UltiMate 3000 UHPLC (Dionex) was coupled to a Q Exactive Plus Mass Spectrometer (QE-MS, Thermo Fisher Scientific) for metabolite profiling. A hydrophilic interaction chromatography method employing an Xbridge amide column (100 × 2.1 mm i.d., 3.5 μm; Waters) was used for polar metabolite separation. The detailed LC method was described previously (42, 43) except that mobile phase A was replaced with water containing 5 mM ammonium acetate (pH 6.8). The QE-MS was equipped with a heated electrospray ionization probe with related parameters set as follows: heater temperature, 120°C; sheath gas, 30; auxiliary gas, 10; sweep gas, 3; spray voltage, 3.0 kV for positive mode and 2.5 kV for negative mode; capillary temperature, 320°C; S-lens, 55; scan range (*m*/*z*), 70 to 900 for positive mode (1.31 to 12.5 minutes) and negative mode (1.31 to 6.6 minutes) and 100 to 1,000 for negative mode (6.61 to 12.5 minutes); resolution, 70,000; automated gain control, 3 × 10^6^ ions. Customized mass calibration was performed before data acquisition. LC-MS peak extraction and integration were performed using commercially available software Sieve 2.2 (Thermo Fisher Scientific). The peak area was used to represent the relative abundance of each metabolite in different samples. The missing values were handled as described previously (43).

### LC-MS/MS analysis of GSH ^13^C isotope incorporation.

Approximately 1 million 769p cells were maintained for 6 hours in RPMI media containing 10% dialyzed FBS, 133 μM glycine, and no serine. Then ^13^C_3_-labeled serine was added to the cells at a final concentration of 300 μM. Twenty-four hours later, the cells were washed once with ice-cold PBS and treated with ice-cold 80% methanol for 30 minutes to extract metabolites including GSH. The extractions were centrifuged at 3,000*g* for 10 minutes, and the supernatants were removed and dried to completion under nitrogen gas. The samples were then resuspended in 100 μL of 0.1% formic acid in double-distilled H_2_O before LC-MS analysis. An aliquot (20 μL) of each sample was loaded onto a 2.1 × 100 mm, 1.7 μm Luna Omega 80 Å reverse-phase column (Phenomenex) at a flow rate of 400 μL/min. A linear gradient of 5%–50% mobile phase B for 10 minutes, then 50%–98% B for 11 minutes with a 1-minute hold, then re-equilibration at initial conditions for 8 minutes using an Exion UHPLC (Sciex) and a flow rate of 100 μL/min was used. The mobile phases were (A) double-distilled H_2_O with 0.1% formic acid and (B) acetonitrile with 0.1% formic acid. The Sciex 5600 Triple-Tof mass spectrometer was used to analyze the GSH ^13^C profile. The IonSpray voltage for positive mode was 5,500 V, and the declustering potential was 100 V. IonSpray GS1/GS2 and curtain gases were both set at 40 psi. The interface heater temperature was 400°C. Eluted compounds were subjected to a time-of-flight survey scan from *m*/*z* 50–1,000. Product ion time-of-flight scans for each possible ^13^C incorporation of GSH were obtained to capture tandem mass spectra ranging from *m*/*z* 308.1 to 318.1, respectively, and were collected over 50-millisecond intervals using a collision energy spread of 10 V with a set collision point of 25 V. Spectra were centroided and de-isotoped by Analyst software version 1.81 TF (Sciex). LC-MS sample data were processed using PeakView Software 2.2 (Sciex) to measure the GSH isotopic patterns and assess the ^13^C incorporation sites using tandem MS data collected for each via product ion scans. The ProteinProspector website (https://prospector.ucsf.edu/prospector/mshome.htm) was used to predict fragmentation patterns for the GSH ^13^C incorporation.

### RT-qPCR and RNA-Seq.

Total RNA from cultured cells or tissues was extracted using Trizol reagent (Invitrogen) and RNA Miniprep kit (Zymo Research). cDNA was synthesized using High-Capacity cDNA Reverse Transcription Kit (Thermo Fisher Scientific). Real-time qPCR was performed using QuantStudio 6 Flex (Thermo Fisher Scientific). For RNA-Seq from cell lines (RXF-control, RXF-L2HGDH) or tissues (normal kidney or low– or high–l-2HG kidney tumors), RNA was extracted followed by rRNA depletion. High-quality total RNA (RIN > 7.0) was paired-end-sequenced using Illumina HiSeq 2500 by HudsonAlpha Institute for Biotechnology, Huntsville, Alabama, USA (https://www.hudsonalpha.org/). After base calls by Illumina bcl2fastq (v2.18.0.12), FASTQ files were aligned to GRCh37 (release 25) reference genome assembly using STAR (v2.7.10a) (https://github.com/alexdobin/STAR). Finally, transcript abundance was calculated using HTSeq-count (v2.0.2) (https://pypi.org/project/HTSeq/), and differential gene expression was analyzed by DESeq2 (https://www.bioconductor.org/packages/release/bioc/html/DESeq2.html).

### Proliferation assays.

Proliferating assay was performed either by cell counting using a hemocytometer or by xCELLigence real-time cell analysis by ACEA Biosciences (Agilent) as instructed by the company. Normalization was performed 24 hours after cell attachment for both the methods.

### Immunoblotting.

For total cell lysates, cell pellet dissolved in DPBS containing 2% (wt/vol) SDS was sonicated briefly and centrifuged (16,000*g*, 10 minutes, room temperature), and the supernatant was collected for analysis. Protein from whole-cell lysates was quantified using a Pierce BCA Protein Assay Kit (Thermo Fisher Scientific). Ten- to twenty-microgram lysates were resolved by 4%–15% SDS-PAGE (Bio-Rad), transferred to PVDF membrane (Bio-Rad), and incubated with primary antibodies following standard protocols. Blots were developed using HRP-conjugated anti-rabbit (45-000-683) or anti-mouse (45-000-679) secondary antibodies (GE HealthCare) and HRP substrate (Millipore WBLUC0500). Primary antibodies used in this study were as follows: anti-L2HGDH (Genetex GTX32695), anti-PHGDH (Cell Signaling 13428S), anti–β-actin (Abcam ab20272), anti-PSAT1 (Proteintech 10501-1-AP), anti-ATF4 (Cell Signaling 11815S), anti-KDM4C (Novus NB110-38884), anti-ALKBH5 (Cell Signaling 80283S), anti-FTO (Proteintech 27226-1-AP), anti-ASNS (Proteintech 14681-1-AP), anti-FLAG (MilliporeSigma F1804), and anti-METTL3 (Cell Signaling 96391S).

### siRNA gene knockdown.

For siRNA-mediated gene knockdown, we followed the Lipofectamine RNAiMAX manufacturer’s protocol (Thermo Fisher Scientific 13778150). Briefly, cells were seeded in a 6-well plate (200,000 cells per well). After 24 hours, the cells were treated with scrambled or gene-targeted siRNA constructs mixed with RNAiMAX in reduced-serum Opti-MEM medium (Thermo Fisher Scientific 31985070). Fifty to sixty hours after the treatment, cells were harvested for analysis. Details of siRNAs used in this study are listed in Supplemental Table 5.

### MTS assay.

Cell viability upon exposure to oxidative stress was measured with a commercial cell viability MTS assay kit (Abcam ab197010). Cells were grown in a 96-well plate and treated with 100 μM *tert*-butyl H_2_O_2_ (Thermo Fisher Scientific) in designated media conditions for 8 hours and then assessed for viability following the kit protocol.

### Xenograft.

Cells were grown in RPMI media followed by trypsinization and washed 3 times in DPBS with centrifugation in between. Cells were mixed in a 1:1 concentration with Matrigel (Corning 356234) and kept on ice until injection. Cells were injected in the flanks of immunocompromised mice (1.5 million cells in OS-RC-2 experiments and 3 million cells in 786-O experiments) as previously described (38).

### ALKBH5 assay.

ALKBH5 enzymatic assay in the presence of l-2HG was assessed by a commercial ALKBH5 chemiluminescent assay kit (BPS Biosciences 79650) following the manufacturer’s protocol. The luminescence signal was measured by GloMax navigator (Promega GM2000).

### Luciferase assay.

pCAX human ATF4 5′-uORF plasmid was provided by Takao Iwawaki (17). *Renilla* plasmid (pRL-SV40P) was a gift from Ron Prywes via Addgene (44). Cells were trypsinized and plated in HBSS with 10% dialyzed FBS for 4–8 hours before firefly luciferase and *Renilla* (internal control) measurements were taken using Promega Dual-Glo Luciferase Assay (Thermo Fisher Scientific).

### Dot blot.

mRNA was isolated from total RNA using a MilliporeSigma/Roche mRNA isolation kit. mRNA concentration was measured using a NanoDrop spectrophotometer (Thermo Fisher Scientific). mRNA was denatured at 65°C for 15 minutes followed immediately by chilling on ice. mRNA was dotted on a nitrocellulose membrane (Bio-Rad). Once the membrane was dried, RNA was cross-linked to the membrane using 120 mJ/cm^2^ density of UV light (UVP Hybrilinker HL-2000). Next, the membrane was washed once with 2× SSC solution (Thermo Fisher Scientific), then incubated with 1× Blot Stain Blue (MilliporeSigma) for 1–2 minutes, and then rinsed several times with 1× TBST (Tris-buffered saline, pH 7.4, plus 0.1% Tween-20) until the background blue was minimized. After imaging of the blue RNA dots, the membrane was blocked with 5% milk for 1 hour followed by overnight incubation with anti-m6A antibody (Cell Signaling; 1:1,000 dilution) at 4°C. After washing (3 times), the membrane was incubated with HRP-conjugated anti-rabbit secondary antibody (GE HealthCare) for 1 hour (1:3,000 dilution), and finally, the image was captured by a phosphorimager (Amersham, GE).

### GSH measurements.

Twenty hours after plating of 300,000 cells in a 6-well plate, media were changed to the corresponding experimental conditions. After 10 hours, cells were harvested, and total GSH pool (GSH plus GSSG) was measured either by the protocol described previously (45) or by the protocol using the Thermo Fisher Scientific Glutathione Colorimetric Detection kit.

### RNA immunoprecipitation, RT-qPCR, and m6A sequencing.

m6A RNA immunoprecipitation was performed following the protocol provided by EpiMark N6-Methyladenosine Enrichment Kit (NEB E1610S). Briefly, mRNA was isolated from total RNA following the manufacturer’s protocol (MilliporeSigma 11741985001). Approximately 250 ng mRNAs were subjected to restricted digestion using NEBNext Magnesium RNA Fragmentation Module (NEB E6150S) (5 minutes, 94°C). mRNA fragments were purified using RNA Clean and Concentrator Kit (Zymo Research R1013). Then, the purified mRNA fragments were subjected to immunoprecipitation using m6A or control antibody–coated Protein G magnetic beads following the manufacturer’s protocol. Immunoprecipitated mRNA fragments were purified, reverse-transcribed, and analyzed by RT-qPCR using *PSAT1*-3′-UTR-F1 and *PSAT1*-3′-UTR-R1 primers. Details of the primers are listed in Supplemental Table 5. m6A RNA sequencing was performed by LC Sciences (Houston, Texas, USA; https://lcsciences.com/) using its proprietary m6A immunoprecipitation cut paired-end sequencing service with Illumina Novaseq6000 platform.

### SELECT assay.

For base-specific quantification of m6A modification, SELECT assay was performed based on the protocol described by Xiao et al. (23). Briefly, a 17 μL reaction mix was set up in ice. The mix contained total RNA (250 ng), SELECT Primer Up (40 nM), SELECT Primer Down (40 nM), dNTP (5 μM), 1× NEB Cutsmart buffer (2 μL), and H_2_O. Each RNA was annealed to Up and Down primers for both the m6A and the control (–6 bp) site. Annealing reaction was performed in a PCR machine (Thermo Fisher Scientific) as follows: 90°C for 2 minutes, 80°C for 2 minutes, 70°C for 2 minutes, 60°C for 2 minutes, 50°C for 2 minutes, 40°C for 12 minutes, 4°C hold. Subsequently, a 3 μL enzyme mixture containing 0.01 U Bst 2.0 DNA polymerase (NEB), 0.5 U SplintR ligase (NEB), and 10 nmol ATP (NEB) was added to a final volume of 20 μL. The mixture was then incubated at 40°C for 40 minutes, 80°C for 20 minutes, and 4°C hold. Two microliters of this SELECT reaction was added to 8 μL of RT-qPCR mix containing 5 μL SYBR Green PCR Master Mix (Thermo Fisher Scientific), 1 μL mix of SELECT qPCR-F and SELECT qPCR-R (200 nM), and 2 μL of nuclease-free H_2_O. RT-qPCR was run in QuantStudio 6 Flex (Thermo Fisher Scientific). Relative m6A levels between experimental conditions were determined using the 1/ΔΔCt method. Details of the primers for the SELECT assay are listed in Supplemental Table 5.

### m6A determination by LC-MS.

Samples were prepared following the protocol described by Mathur et al. with some modifications (46). Briefly, 500 ng purified mRNAs from RCC cells were subjected to nuclease P1 (NEB) digestion in 35 μL reaction volume at 37°C for 2 hours; 2 μL of ZnCl_2_ (stock 0.1 M) and 3 μL PCR-grade H_2_O were added to the digestion reaction to increase the volume to 40 μL. Next, 2 μL NH_4_HCO_3_ (stock 2 M) and 1 U alkaline phosphatase (MilliporeSigma) were added to the reaction and incubated for 2 hours at 37°C. Finally, 1 μL 1.2 M HCl was added to neutralize the solution. The samples were vortexed and then centrifuged for 30 minutes at 16,000*g* at 4°C. The supernatants were collected. LC-MS analysis was used to quantify m6A and unmodified adenosine levels using standard curves generated for each nucleoside.

### Statistics.

Statistical analyses were carried out using GraphPad Prism 9 and SAS 9.4 software. Comparisons between groups for statistical significance were performed by 2-tailed *t* test with *P* less than 0.05 for significance unless otherwise specified. For multiple-group comparisons and repeated measures, 2-tailed ANOVA was performed with the indicated post hoc tests.

### Study approval.

All mouse studies were approved by the IACUC of the University of Alabama at Birmingham. Mice were maintained in standard care and were euthanized at predetermined experimental time points or at the first signs of morbidity according to the standards by the IACUC. All human samples analyzed were acquired from the Cooperative Human Tissue Network and were provided to the investigators in a deidentified manner.

### Data availability.

The published article includes all data sets generated or analyzed during this study. They are included in Supplemental Tables 1–4 and the Supporting Data Values file. Sequencing data (RNA-Seq and m6A-Seq) were deposited in the NCBI’s Gene Expression Omnibus database (GEO GSE228082).

## Author contributions

GJB, AK, and SS participated in the conception and design of the study, and in writing. HFD and JEM assisted with experimental design. AK, GJB, HN, SK, ES, HW, JL, YH, RK, MP, AJF, JWL, DR and NHM acquired data. JL, JMT, WJP, JWL, DR, DA, RR, SB, and DKC assisted with data analysis. Co–first authorship order was based on contribution to manuscript formatting.

## Supplementary Material

Supplemental data

Unedited blot and gel images

Supplemental table 1

Supplemental table 2

Supplemental table 3

Supplemental table 4

Supplemental table 5

Supporting data values

## Version 1 05/14/2024

In-Press Preview

## Version 2 07/01/2024

Electronic publication
