# Supplementary material for: l-2-Hydroxyglutarate remodeling of the epigenome and epitranscriptome creates a metabolic vulnerability in kidney cancer models
Source: J Clin Invest. 2024 May 14;134(13):e171294. doi: 10.1172/JCI171294 (PMC11213505; doi:10.1172/JCI171294)
Supplement: Supplemental data [file jci-134-171294-s015.pdf]

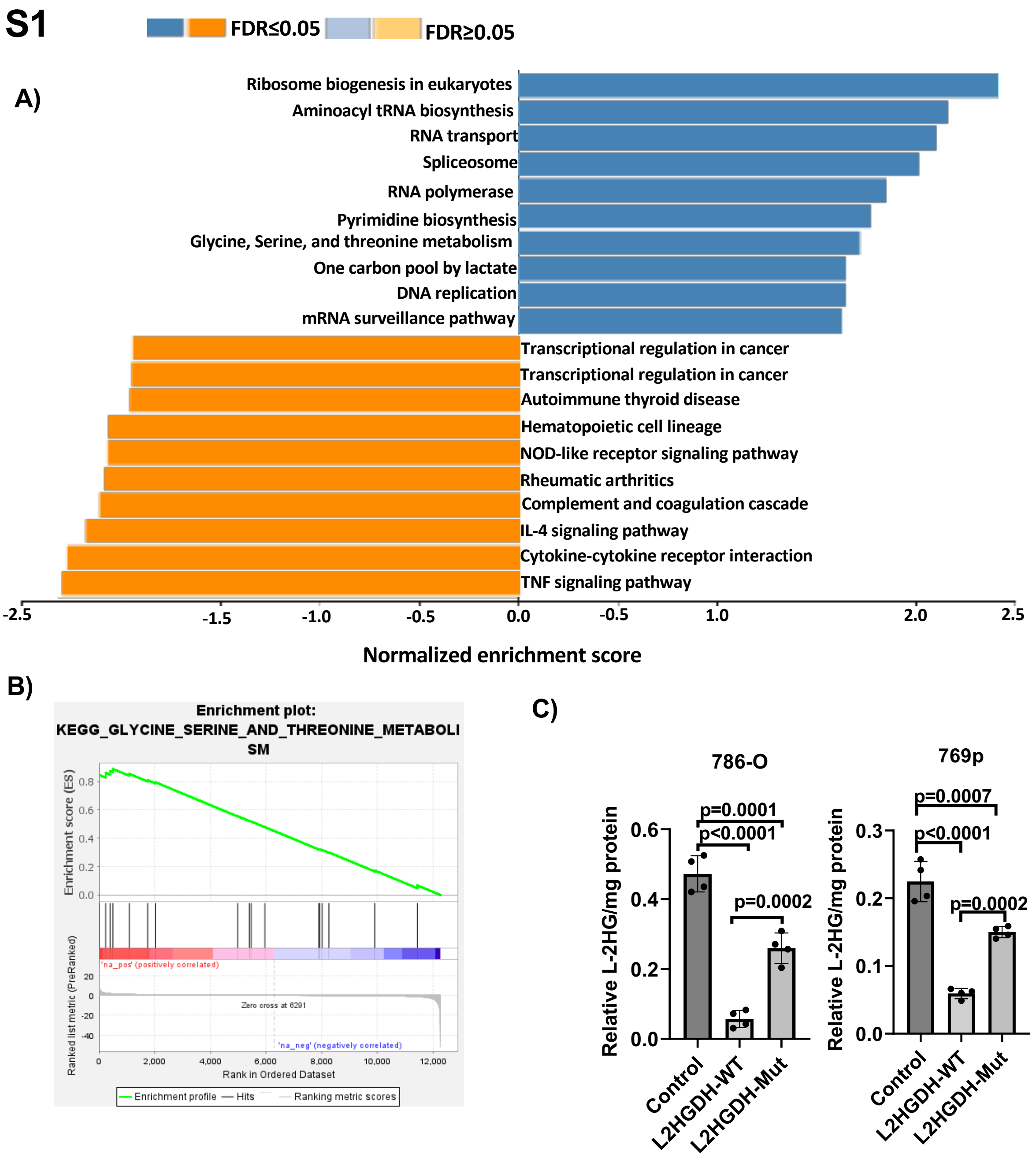

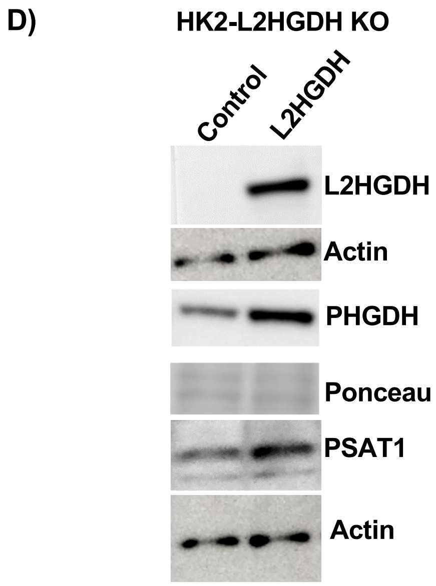

A) Gene ontology enrichment analysis of genes with increased (blue) or decreased (orange) expression upon L2HGDH restoration in RXF-393 cells. B) Enrichment plot for glycine/serine and threonine metabolism in RXF-393 cells restored with L2HGDH. The normalized enrichment score (NES) and FDR for the plot are 2.337 and 0.0, respectively. C) MS-analysis of L-2HG metabolite in 786-O and 769p cells harboring the indicated constructs (X-axis). Data represented as mean ± SD from n=4 biological replicates. Data were analyzed by one-way ANOVA followed by post hoc Tukey's honestly significant difference test. ANOVA p-value for both the cell lines is <0.0001(F=100.4 for 786-O and F=81.15 for 769p). Post hoc analysis p-values are demonstrated in the figures.
D) Immunoblot analysis of L2HGDH KO HK2 cells re-expressing either the control vector or L2HGDH construct.

S2

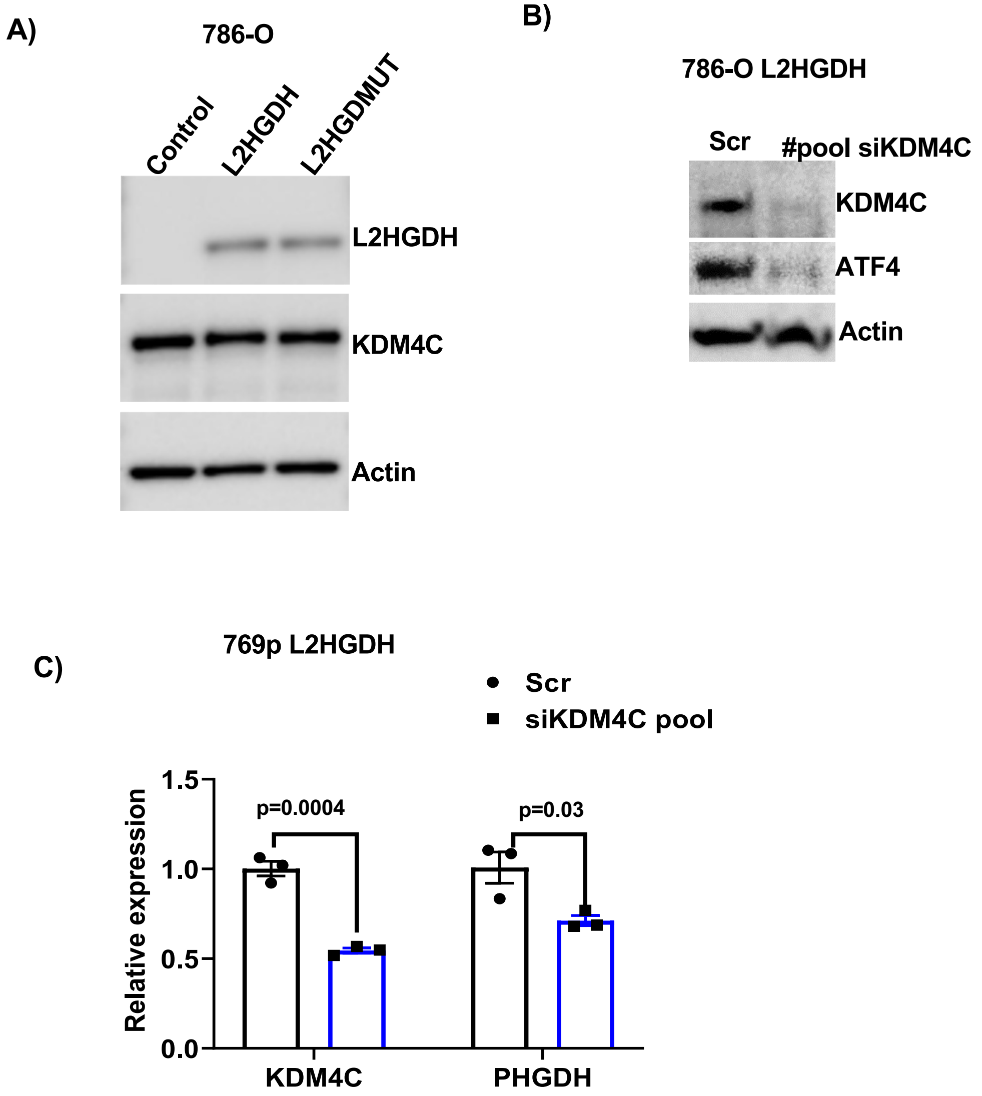

A) Immunoblot analysis of 786-O cells stably expressing either control vector or L2HGDH wild type (WT) or L2HGDH mutant (MUT). Actin was used as the loading control.
B) Immunoblot analysis of 786-O L2HGDH cells treated with either scrambled siRNA (Scr) or siRNA pool targeting KDM4C mRNA for 55 hrs. Actin was used as a loading control.
C) qPCR analysis of *KDM4C* and *PHGDH* mRNA expressions (normalized to *RPLPO*) from 769p L2HGDH cells treated with either scrambled siRNA (Scr) or siRNA pool targeting KDM4C for 55 hrs.

S3

A)

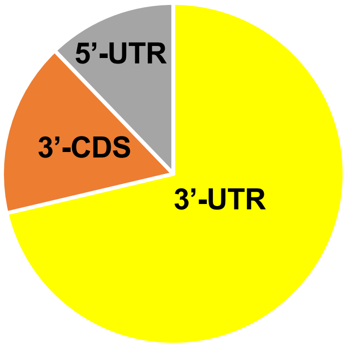

B)

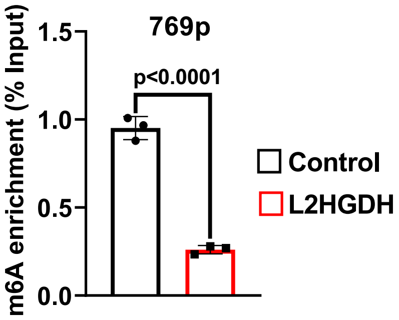

A) The distribution of mRNA m6A peaks in high L-2HG RCC cells.
B) m6A-IP qRT-PCR was used to assess m6A enrichment in the PSAT1 3'UTR from 769p cells stably transduced with the indicated vectors. PSAT1-1F/1R primer pair was used. Data are represented as mean ± SD from n=3 biological replicates.

S4

A) B)

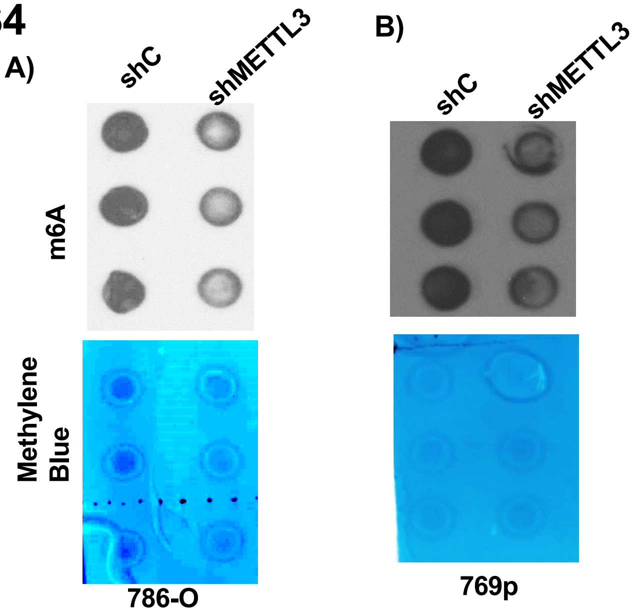

C) PSAT1

>chr9:78328990-78329497
PSAT1
GGCATCCGGGCCTCTCTGTATAATGCTGTCACAATTGAAGACGTTCAGAAGCTGGCCGCCTTCATGAAAAAATTTTTGGAG
ATGCATCAGCTATGAACACATCCTAACCAGGATATACTCTGTTCTTGAACAACATACAAAGTTTAAAGTAACTTGGGGATGG
CTACAAAAAGTTAACACAGTATTTTTCTCAAATGAACATGTTTATTGCAGATTCTTCTTTTTTGAAAGAACAACAGCAAAAC
ATCCACAACTCTGTAAAGCTGGTGGGACCTAATGTCACCTTAATTCTGACTTGAACTGGAAGCATTTTAAGAAATCTTGTTG
CTTTTCTAACAAATTCCCGCGTATTTTGCCTTTGCTGCTACTTTTTCTAGTTAGATTTCAAACTTGCCTGT**GGACT**TAATAATG
CAAGTTGCGATTAATTATTTCTGGAGTCATGGGAACACACAGCACAGAGGGTAGGGGGGCCCTCTAGGTGCTGAATCTAC
ACATCTGTGGGGTCTC

D)

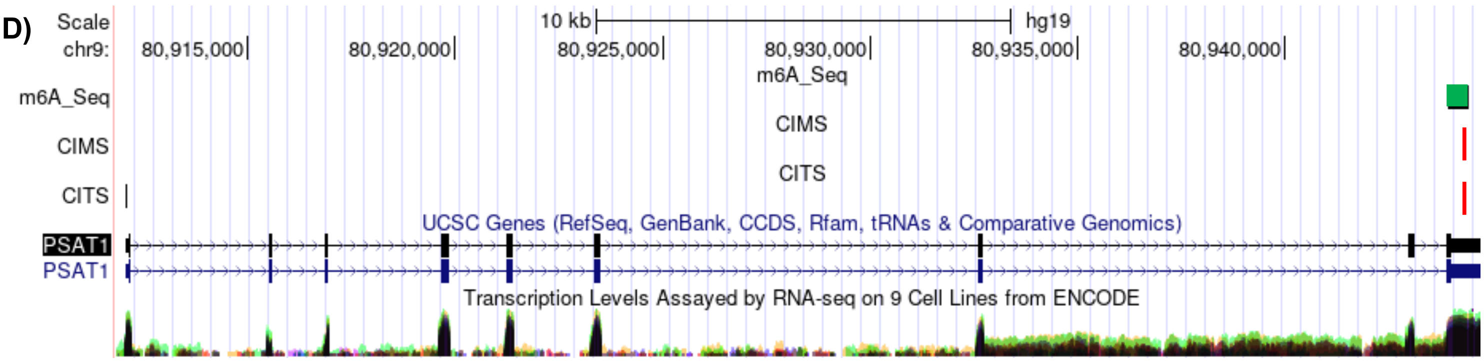

E) 786-O PSAT1 WT

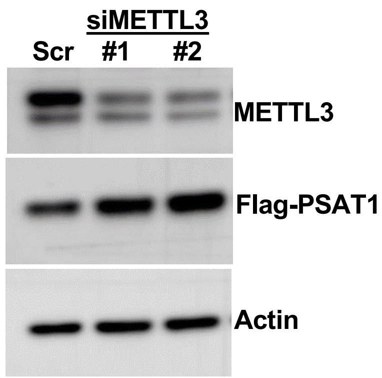

A, B) The level of m6A modification was determined by dot blot of 100 ng of mRNA isolated from 786-O (A) and 769p (B) cells (n=3 biological replicates) stably expressing either control shRNA (shC) or shMETTL3. Methylene blue blot serves as the loading control.
C) DRACH motif (GGACT) is highlighted in the PSAT1-3'UTR region.
D) PSAT1 track on hg19 genome assembly. m6A-modified sites (red lines) from two mi-CLIP data sets (CITS & CIMS) reported by Linder *et al*., (2015) in HEK 293T cells. m6A-enriched region (green bar) present in the 3'-UTR of *PSAT1* in high L-2HG RCC cells.
E) 786-O cells were stably transduced with FLAG-tagged PSAT1 (WT) construct. Cells were transiently transfected (52 hrs) with control siRNA (Scr) or siRNAs targeting METTL3 (#1, #2) followed by immunoblot analysis. Actin was used as the loading control.

S5

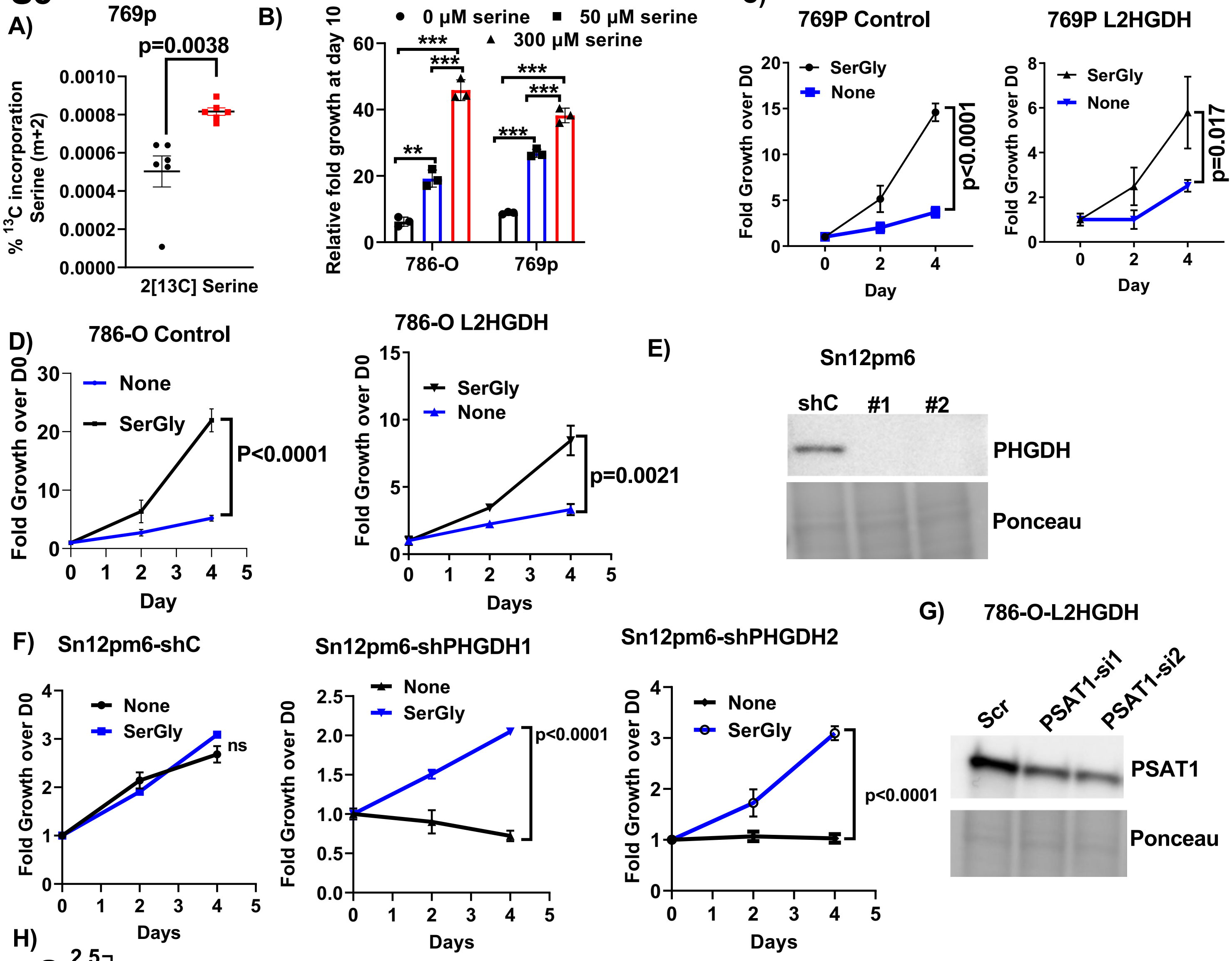

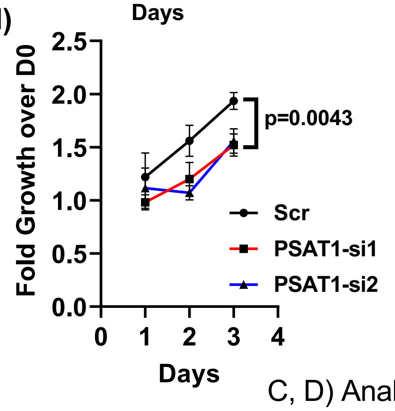

A) LC-MS metabolic flux analysis was used to quantitate 13C labeled serine (m+2) in 769p cells cultured with universally labeled glucose [U-13C]. Data are represented as mean ± SD from n=6 biological replicates. B) Relative fold growth at day 10 for 786-O and 769p cells feeding with MEM media containing indicated concentrations of serine along with 10 μM glycine. Data shown here mean ± SD from n=3 biological replicates. Data were analyzed by one-way ANOVA followed by post hoc Tukey's honestly significant difference test. ANOVA p-value is <0.0001 for both the cell lines. Post hoc analysis p-values are demonstrated in the figures ***p<0.0001, **p<0.005.

C, D) Analysis of relative proliferation of 769p (B) and 786-O (C) cells stably transduced with the indicated vector (left panel- control; right panel- L2HGDH) in media with or without Ser/Gly. Each data point is presented as mean ± SD from n=3 biological replicates. *p<0.05, ns-not significant.

E) Immunoblot of Sn12pm6 cells stably expressing either control shRNA (shC) or two shRNAs (#1, #2) targeting *PHGDH* mRNA. Actin was used as the loading control.

F) Analysis of relative proliferation of Sn12pm6 cells expressing the indicated vector (left panel control shRNA [shC]; middle and right panels- shRNAs targeting *PHGDH*) in media with or without Ser/Gly. Each data point is presented as mean ± SD from n=3 biological replicates. Repeated measures analysis was conducted, and the p-values reflect Tukey's post-hoc comparisons.

G, H) Immunoblot (G) of 786-O L2HGDH cells treated with scrambles (scr) or two siRNA constructs targeting *PSAT1* mRNA and analyzing their proliferation (H) in limited serine conditions. Data shown here mean ± SD from n=3 biological replicates and ANOVA p-value is mentioned in the figure with an F value of 15.45.

S6

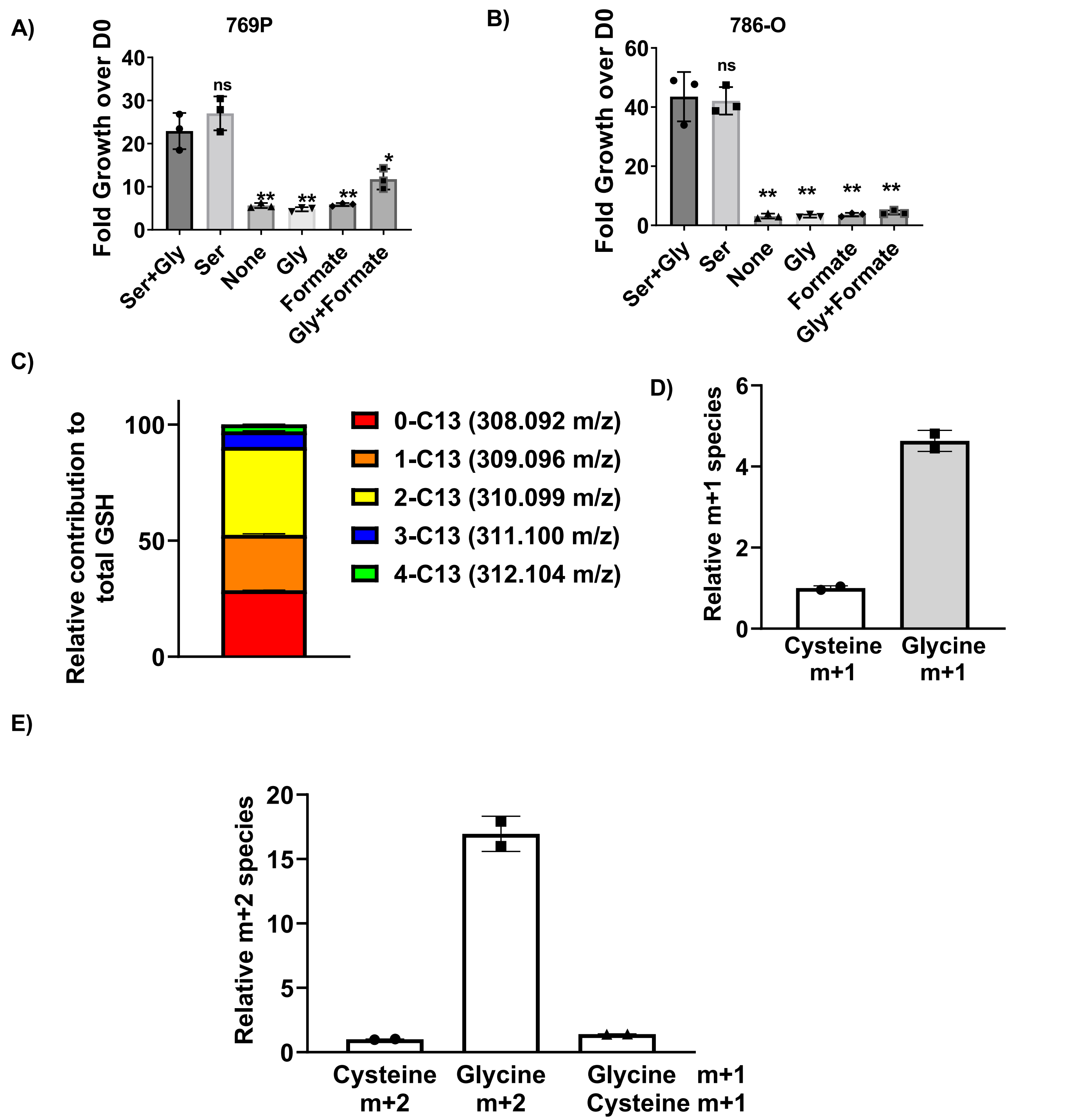

A, B) Analysis of relative proliferation of 769p (A) and 786-O cells (B) grown in RPMI media with the indicated supplements. None denotes cells cultured without serine/glycine. Data presented are mean ± SD from n=3 biological replicates. **p<0.0.005, *p<0.05, ns-not significant. (C) MS/MS Analysis of C13 labeled-GSH species from 769p cells fed with 300 μM 13C3 labeled serine for 24 hrs after being maintained in serine-deprived RPMI media containing 133 μM glycine and 10% dialyzed FBS. D, E) Relative levels of (m+1) (D) and (m+2) (E) massed cysteine and glycine species in 1-C13 and 2-C13 glutathione pools, respectively. Data presented are mean ± SD from n=2 biological replicates.

S7

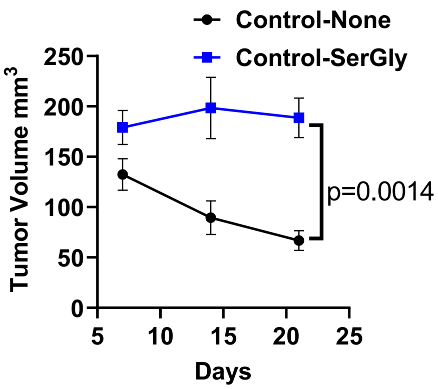

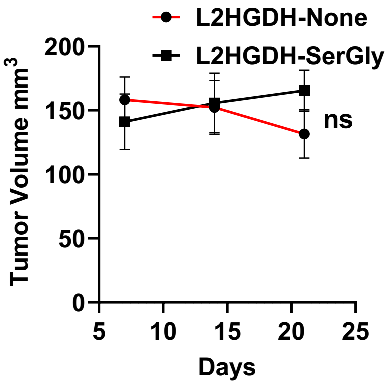

OS-RC-2 (+/- L2HGDH) xenograft growth with or without (none) serine and glycine diet over the course of three weeks. After the average tumor size had reached 100 mm3, mice were randomly distributed into two groups (n=8, each group). Data points are shown here after the diet switch. Data presented as mean ± SEM. Repeated measures analysis was conducted, and the p-values reflect Tukey's post-hoc comparisons.
