# Supplementary material for: l-2-Hydroxyglutarate remodeling of the epigenome and epitranscriptome creates a metabolic vulnerability in kidney cancer models
Source: J Clin Invest. 2024 May 14;134(13):e171294. doi: 10.1172/JCI171294 (PMC11213505; doi:10.1172/JCI171294)

Full unedited gel for Figure 1E

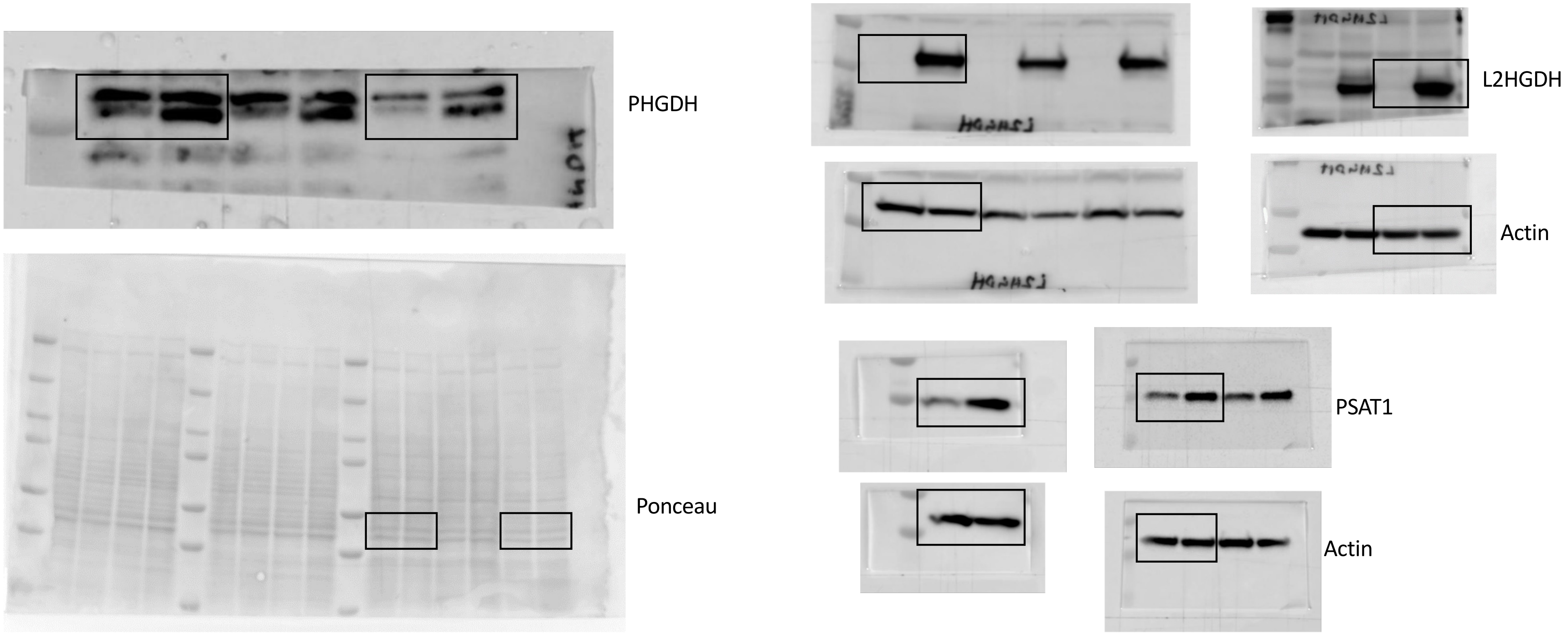

Full unedited gel for Figure 1F

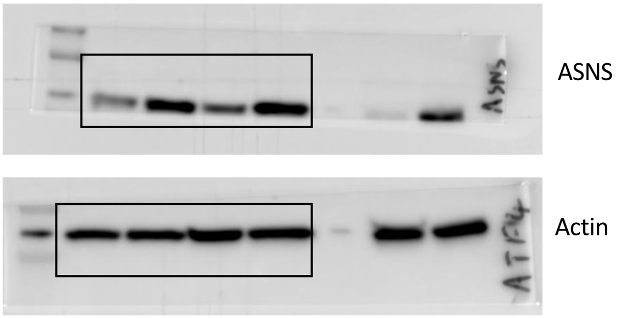

Full unedited gel for Figure 1G

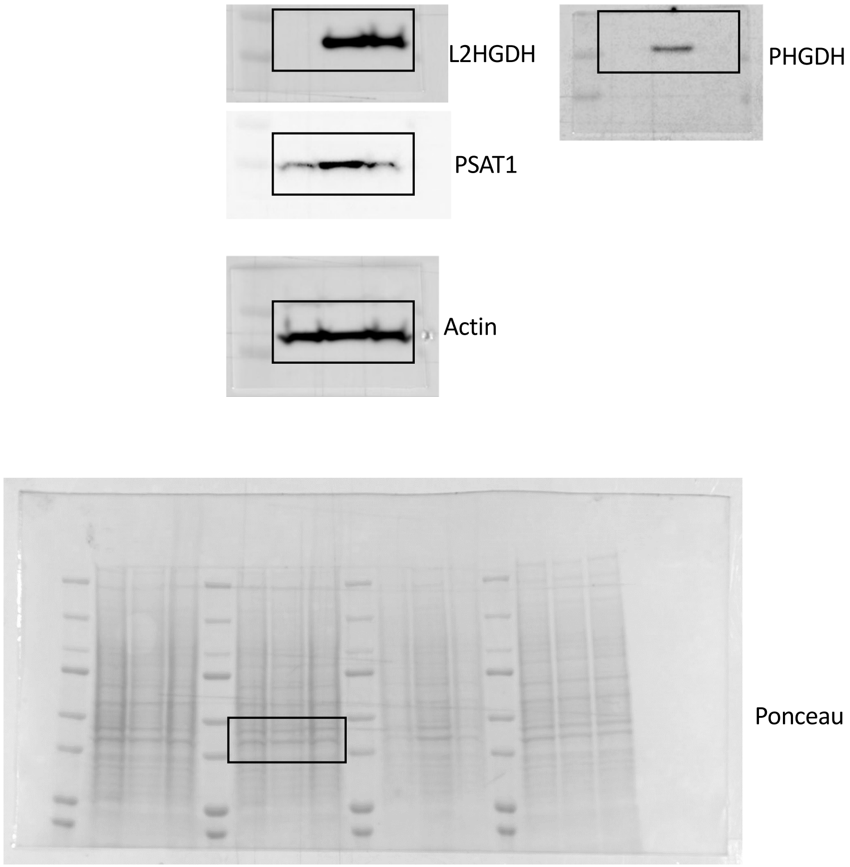

Full unedited gel for Figure 1H

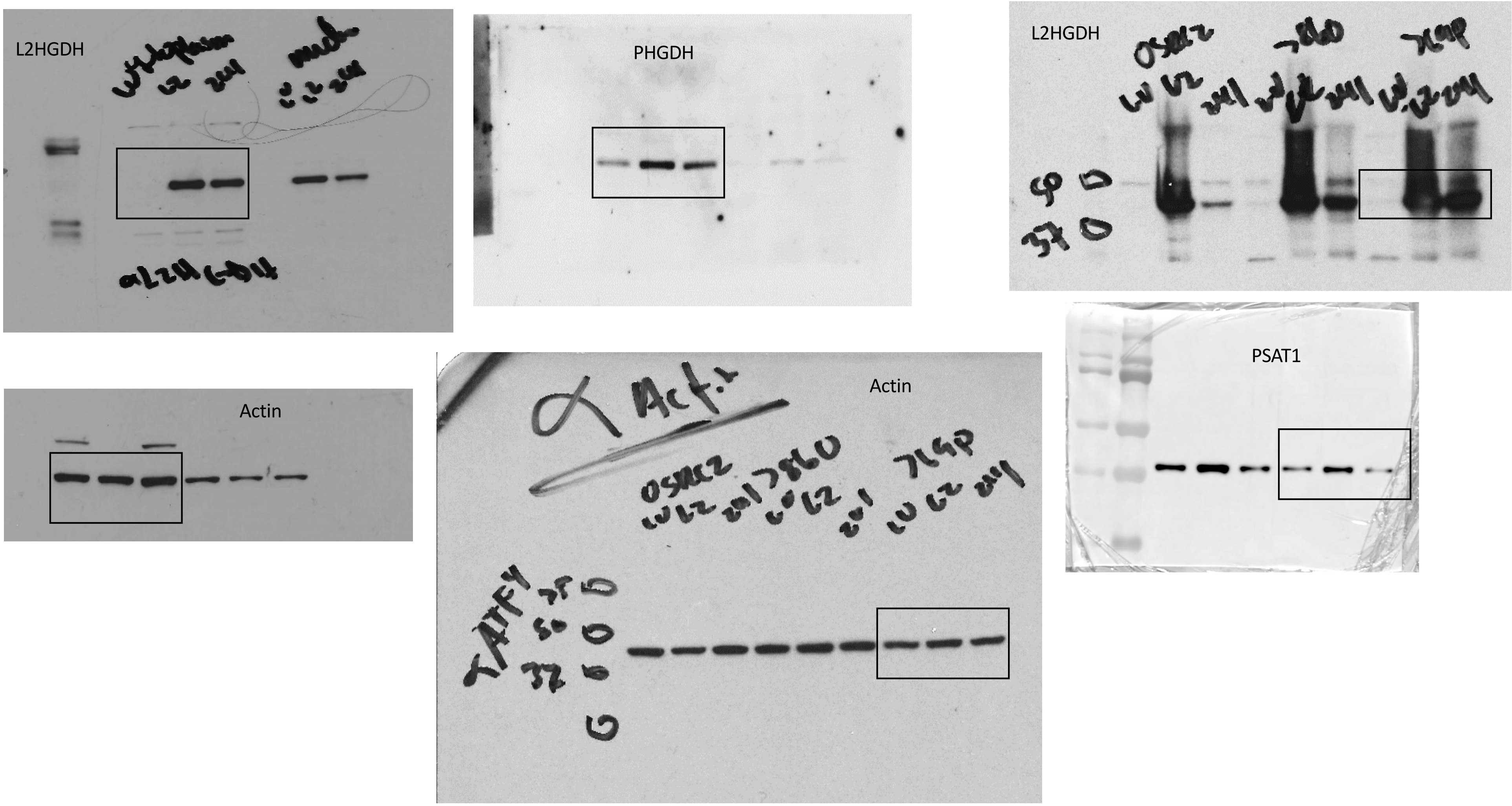

Full unedited gel for Figure 1K

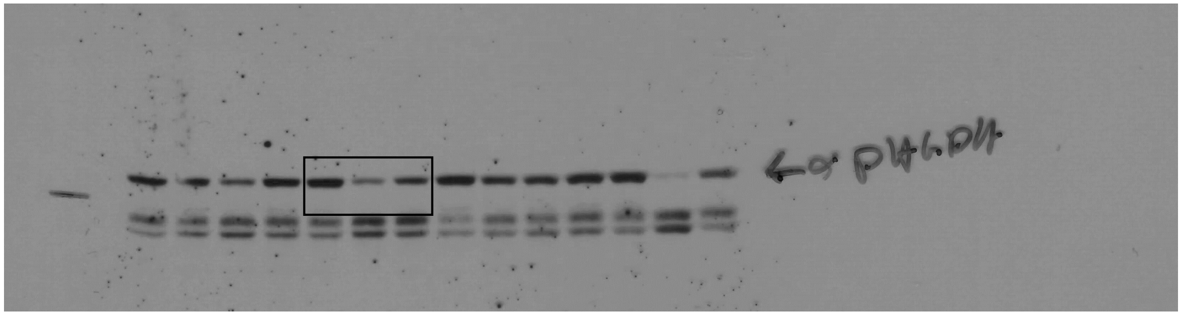

PHGDH

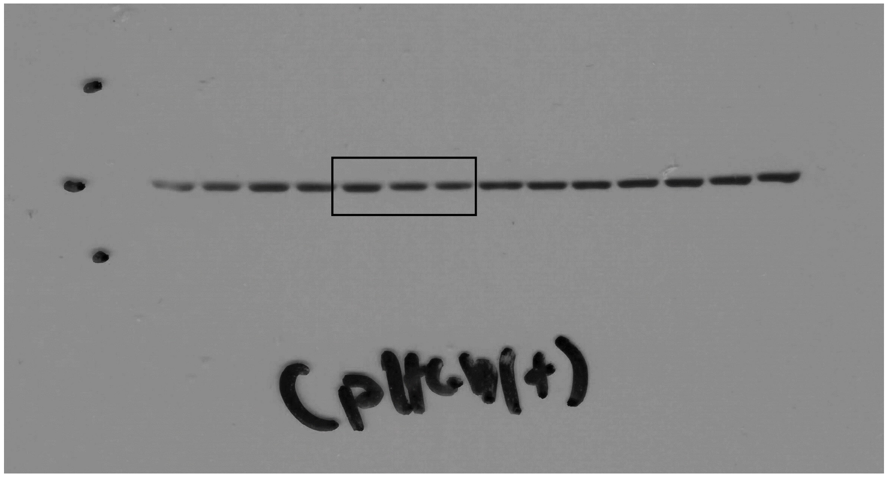

Actin

Full unedited gel for Figure 2A

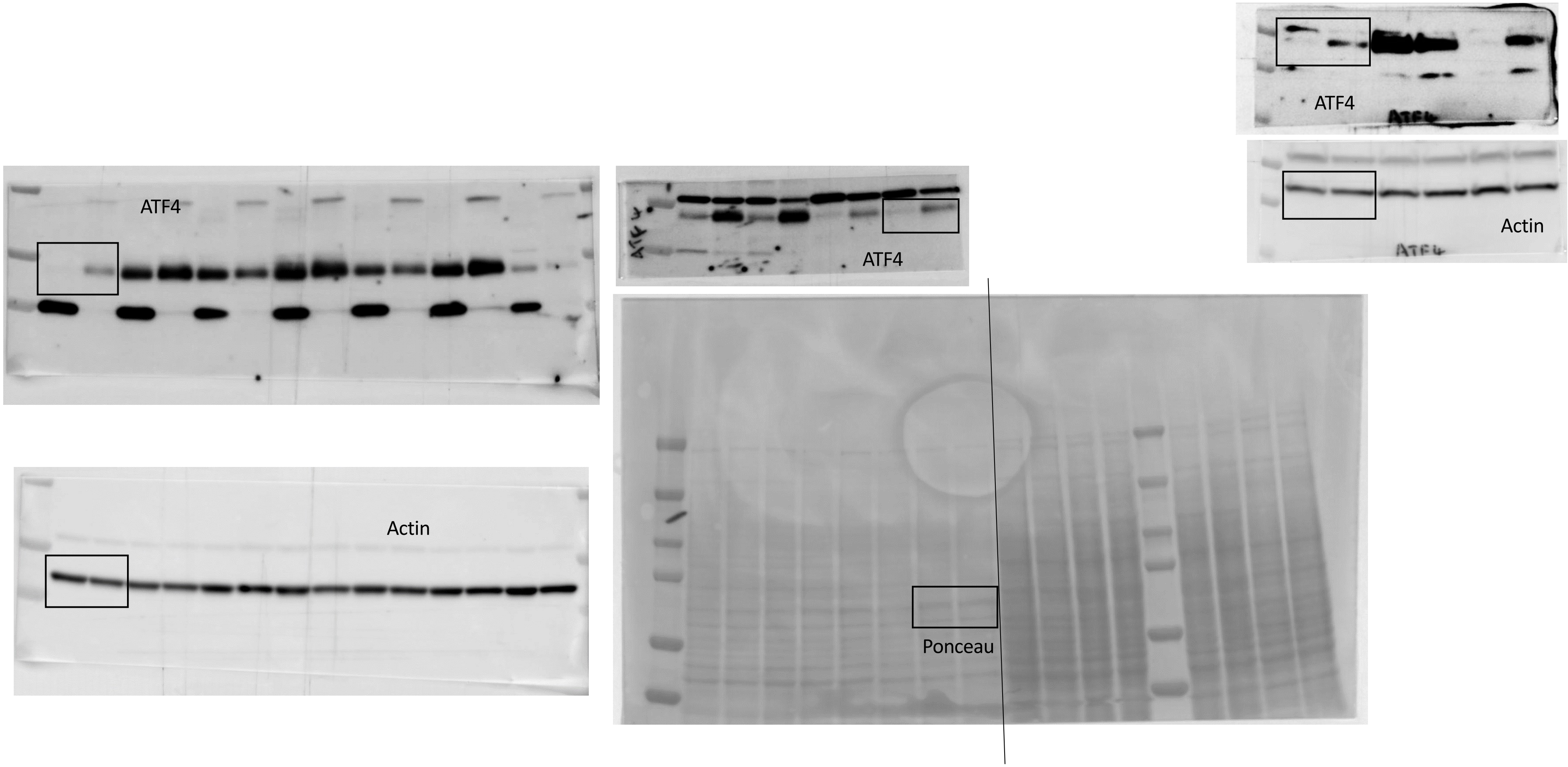

Full unedited gel for Figure 2B

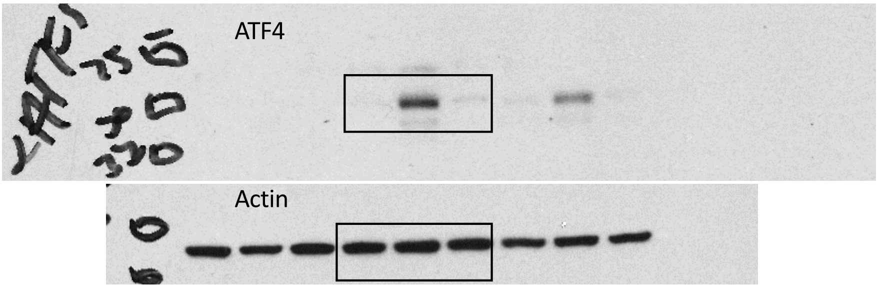

Full unedited gel for Figure 2C

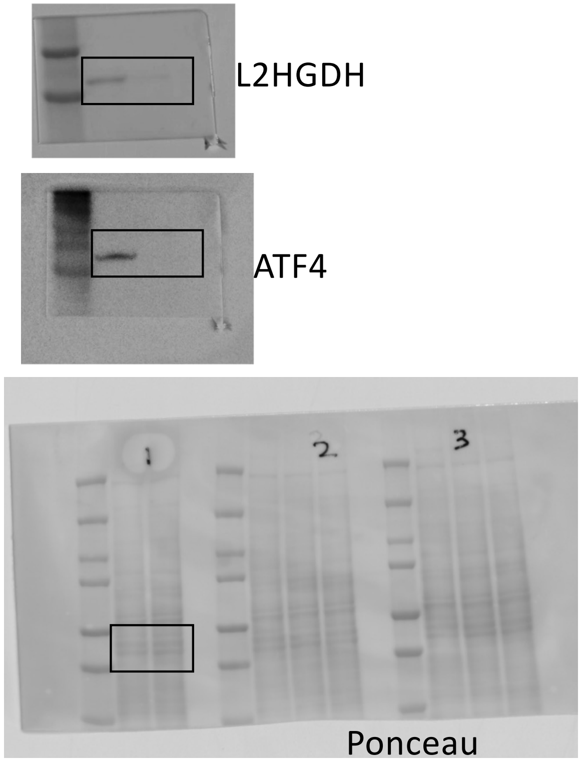

Full unedited gel for Figure 2D

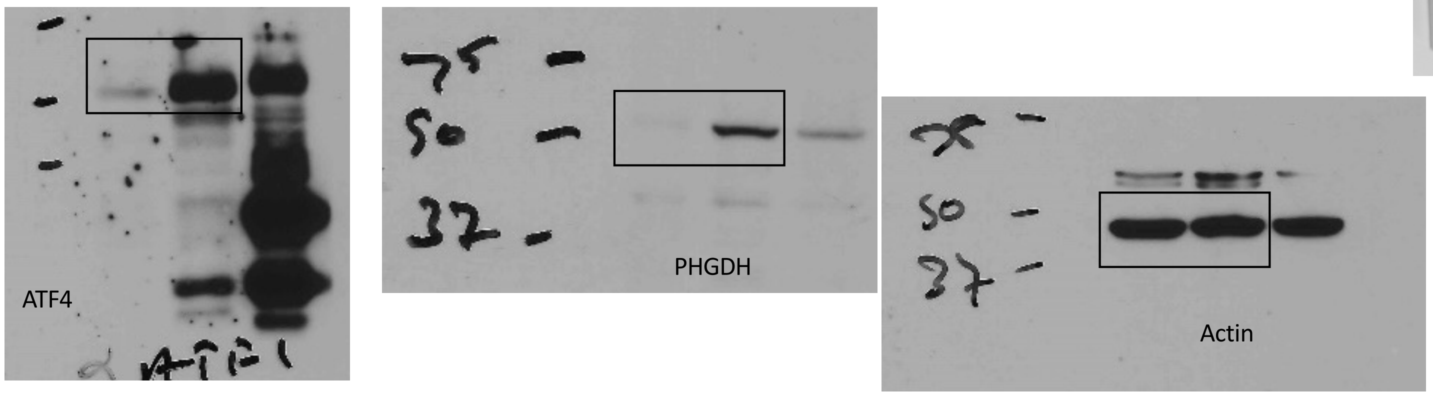

Full unedited gel for Figure 2F

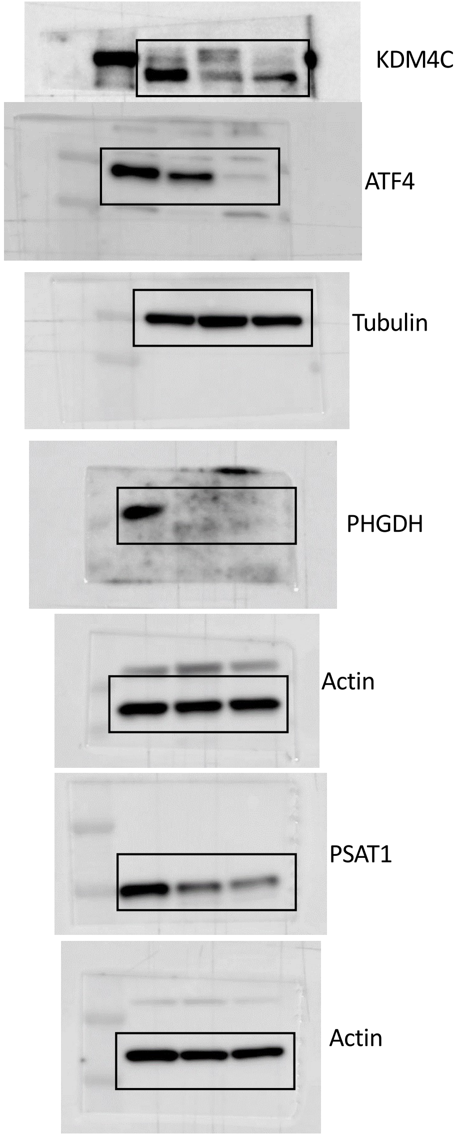

Full unedited gel for Figure 2G

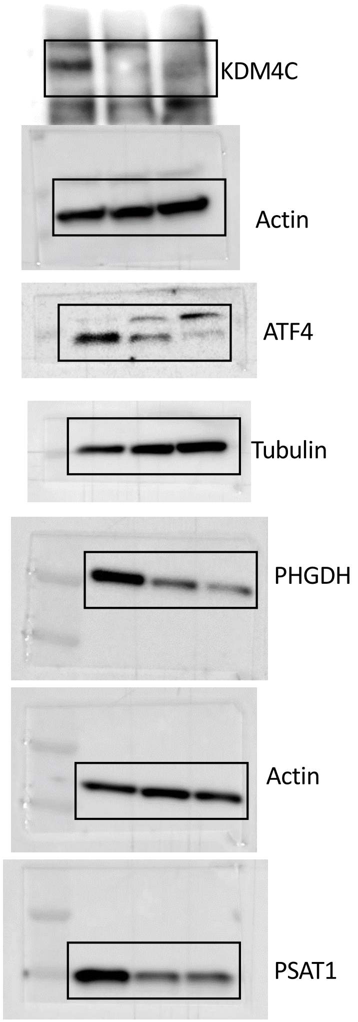

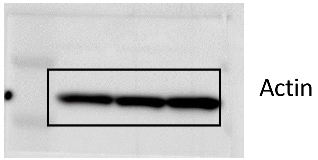

Full unedited gel for Figure 3A

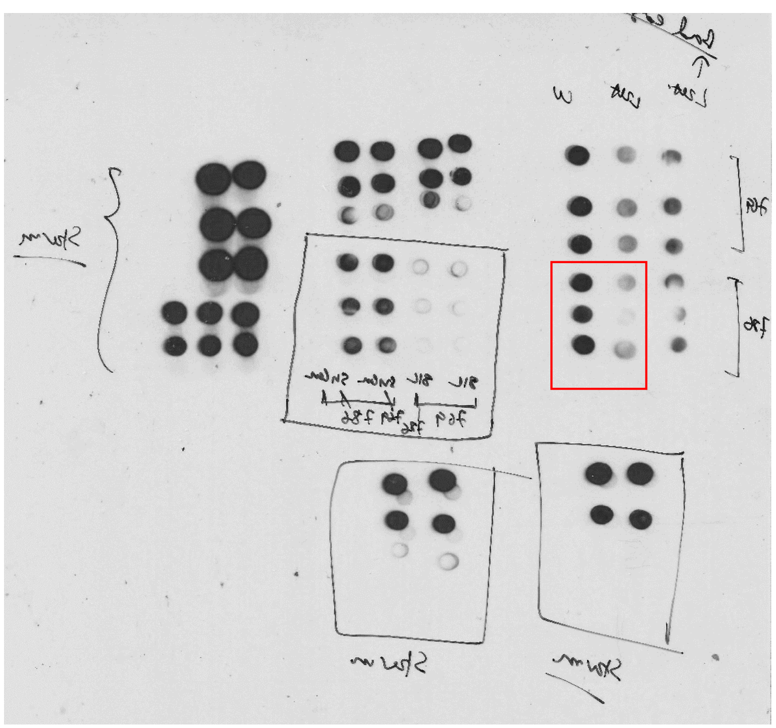

m6A

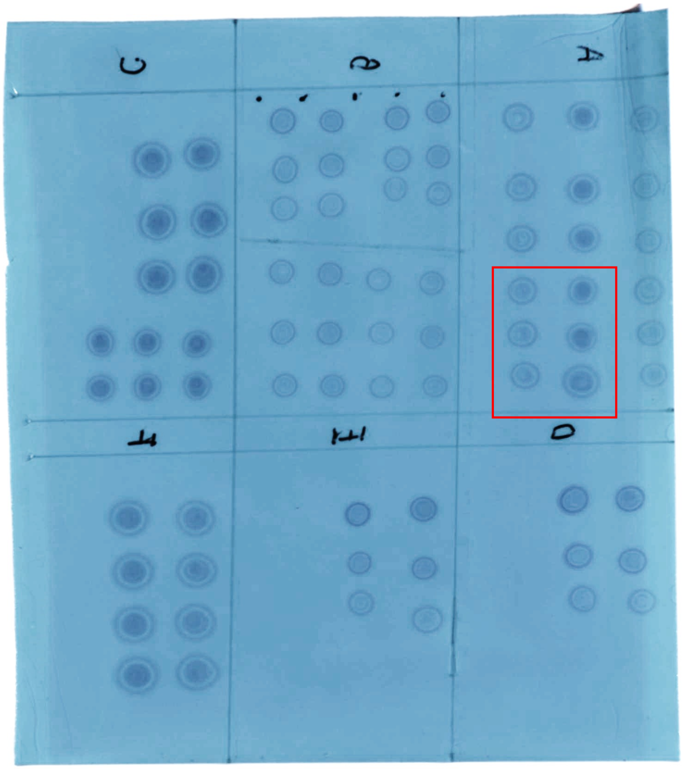

Methylene Blue

Full unedited gel for Figure 3B

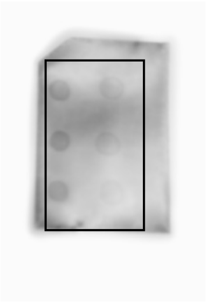

m6A

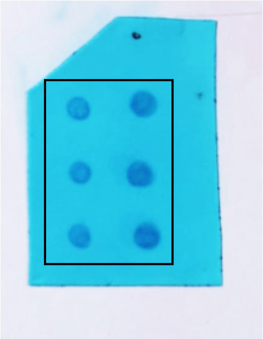

Methylene Blue

Full unedited gel for Figure 3E

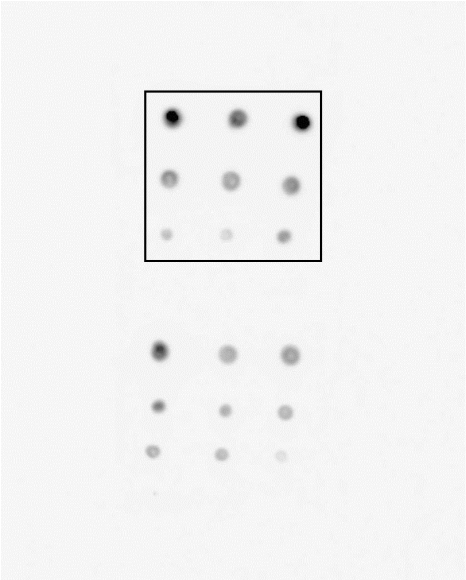

m6A

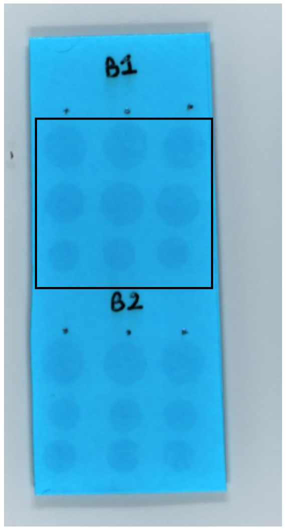

Methylene Blue

Full unedited gel for Figure 3G

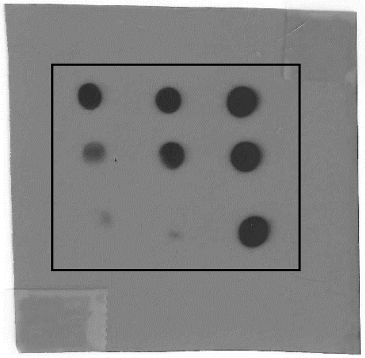

m6A

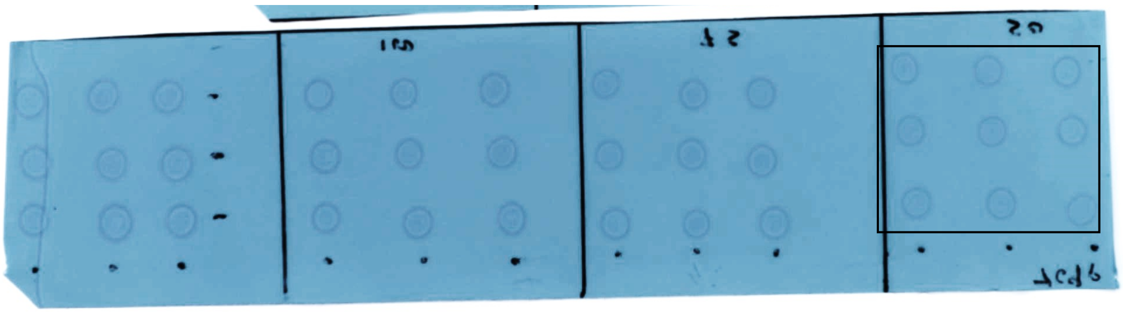

Methylene Blue

Full unedited gel for Figure 4A

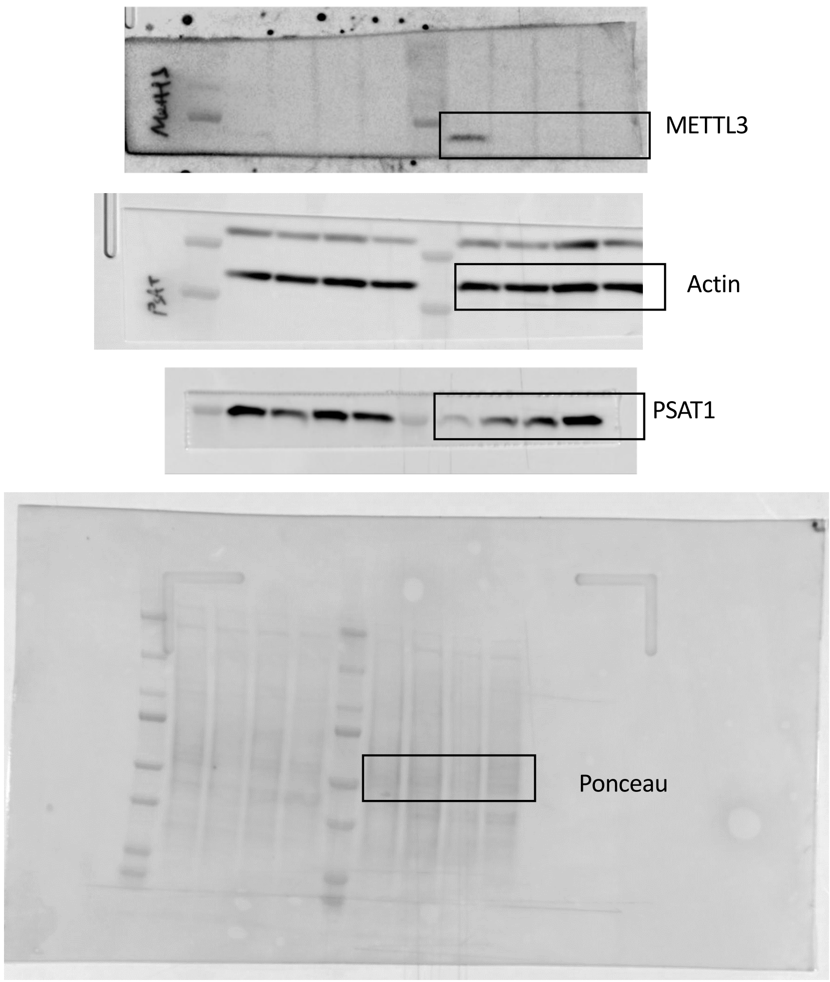

Full unedited gel Figure 4B

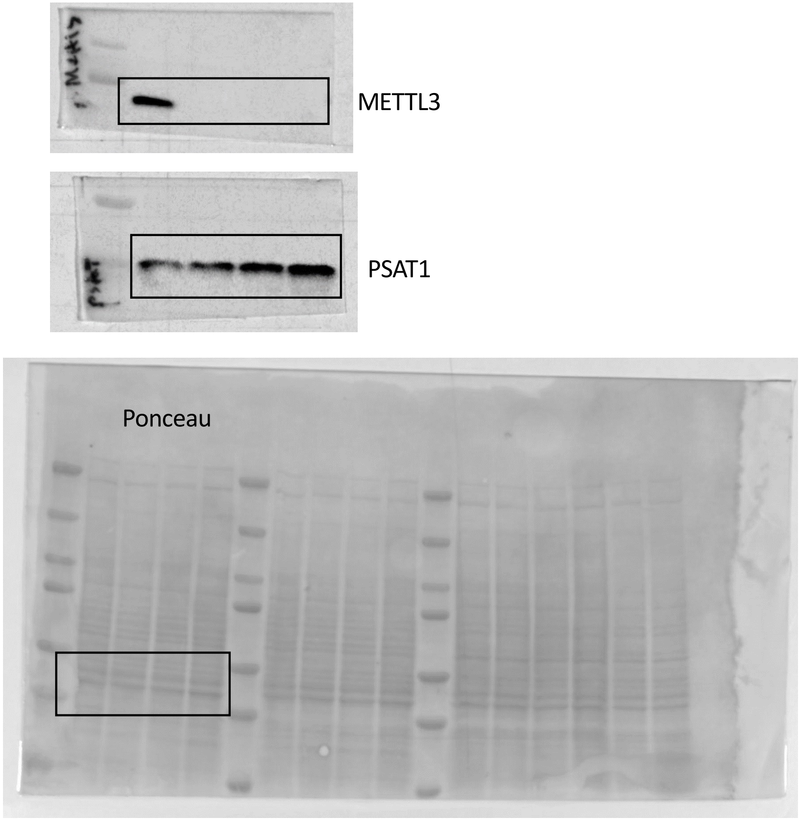

Full unedited gel for Figure 4D

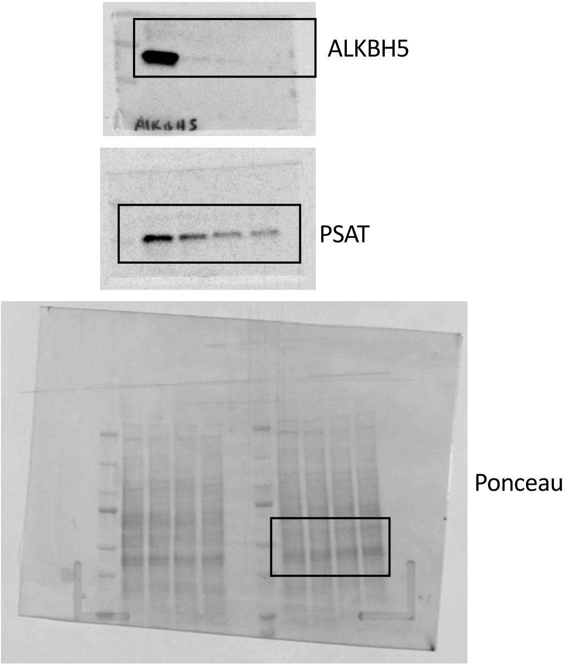

Full unedited gel for Figure 4E

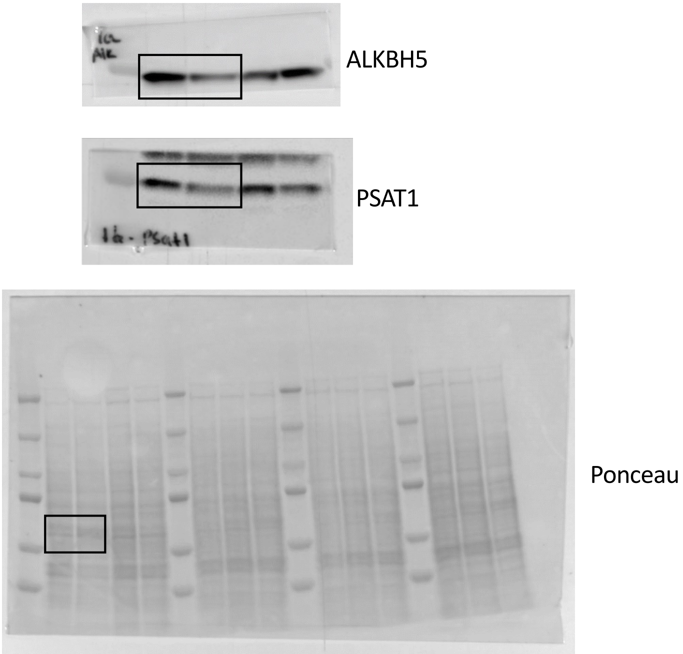

Full unedited gel for Figure 4F

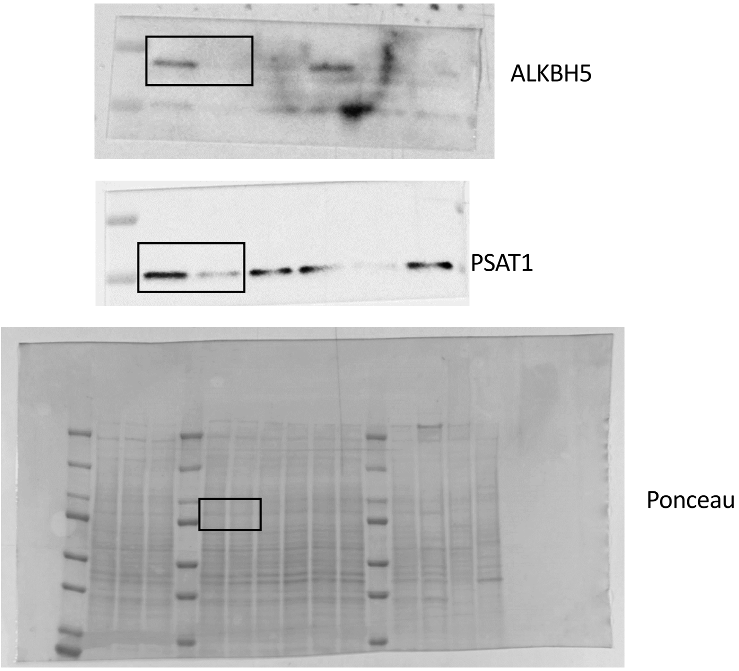

Full unedited gel for Figure 4G

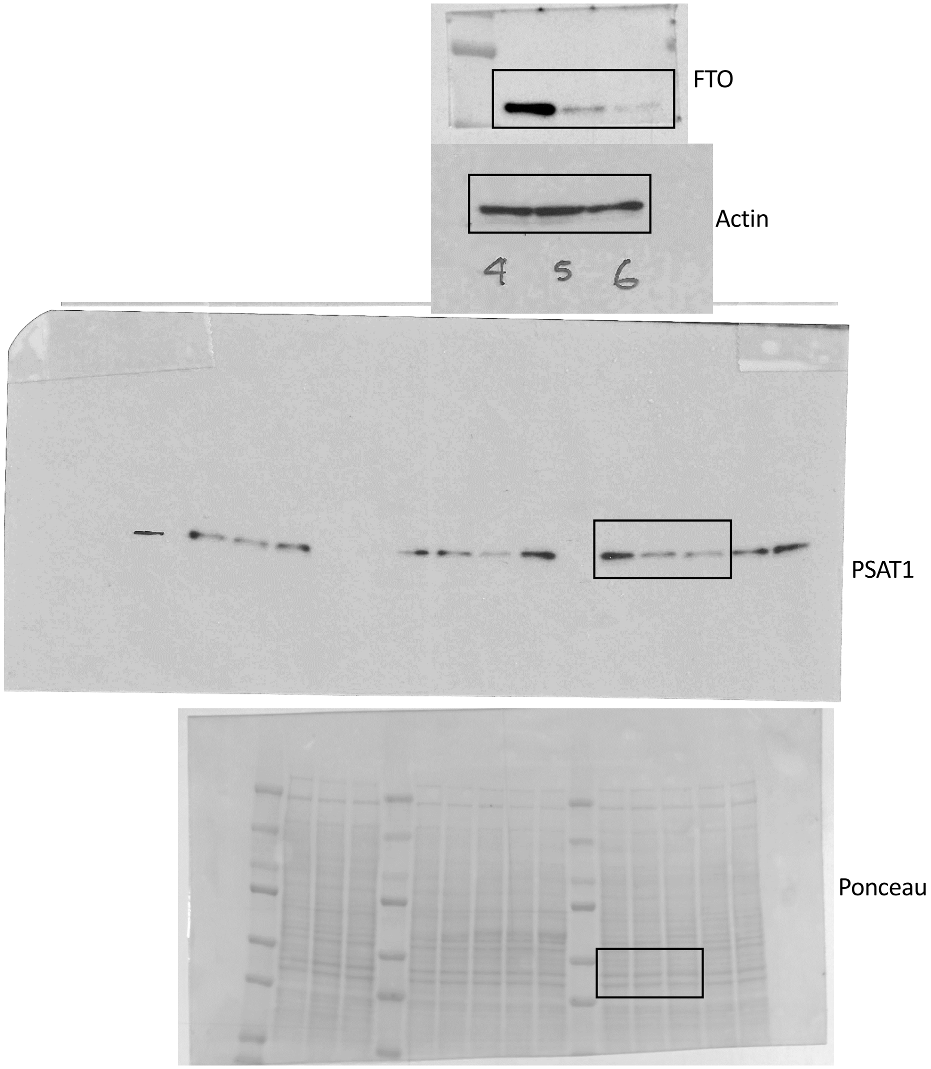

Full unedited gel for Figure 4K

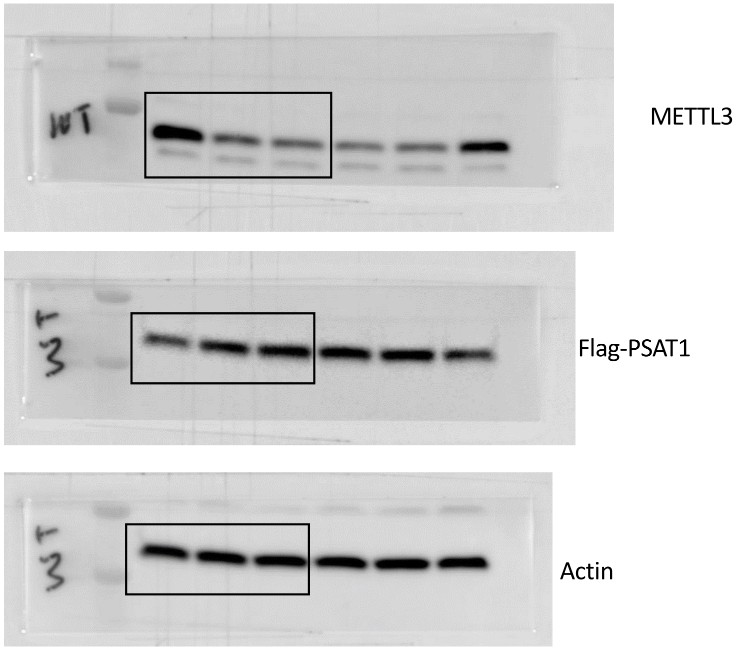

Full unedited gel for Figure 4M

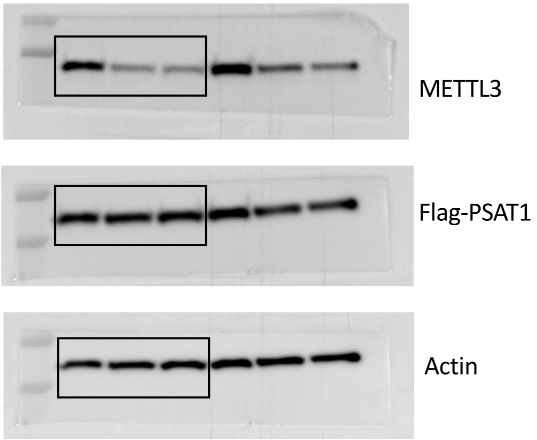

Full unedited gel for Figure 5E

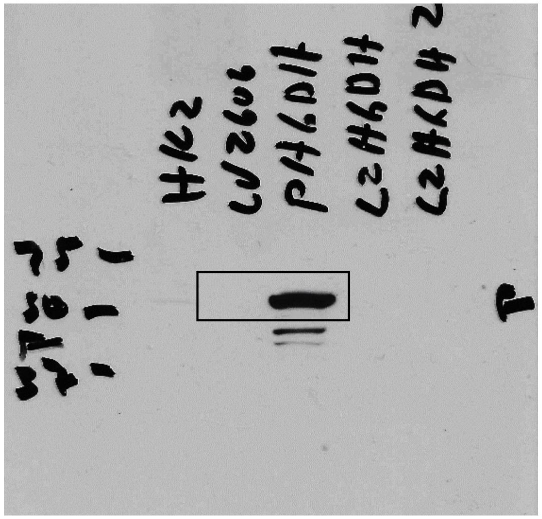

PHGDH

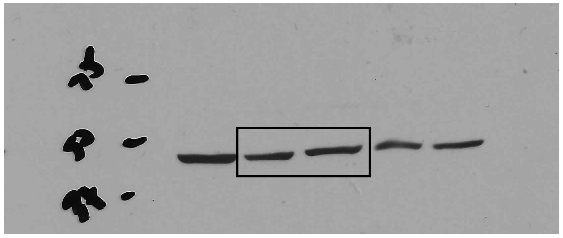

Actin

Full unedited gel for Figure 5H

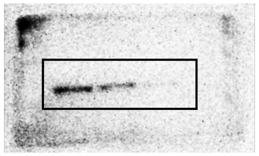

L2HGDH

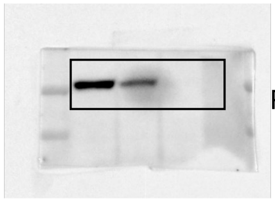

PHGDH

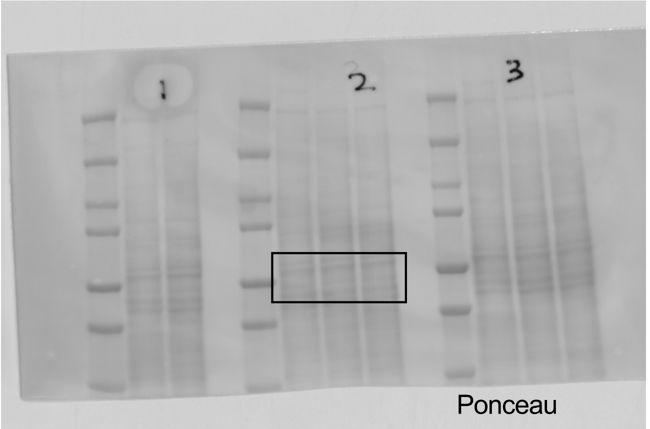

Full unedited gel for Figure 7D

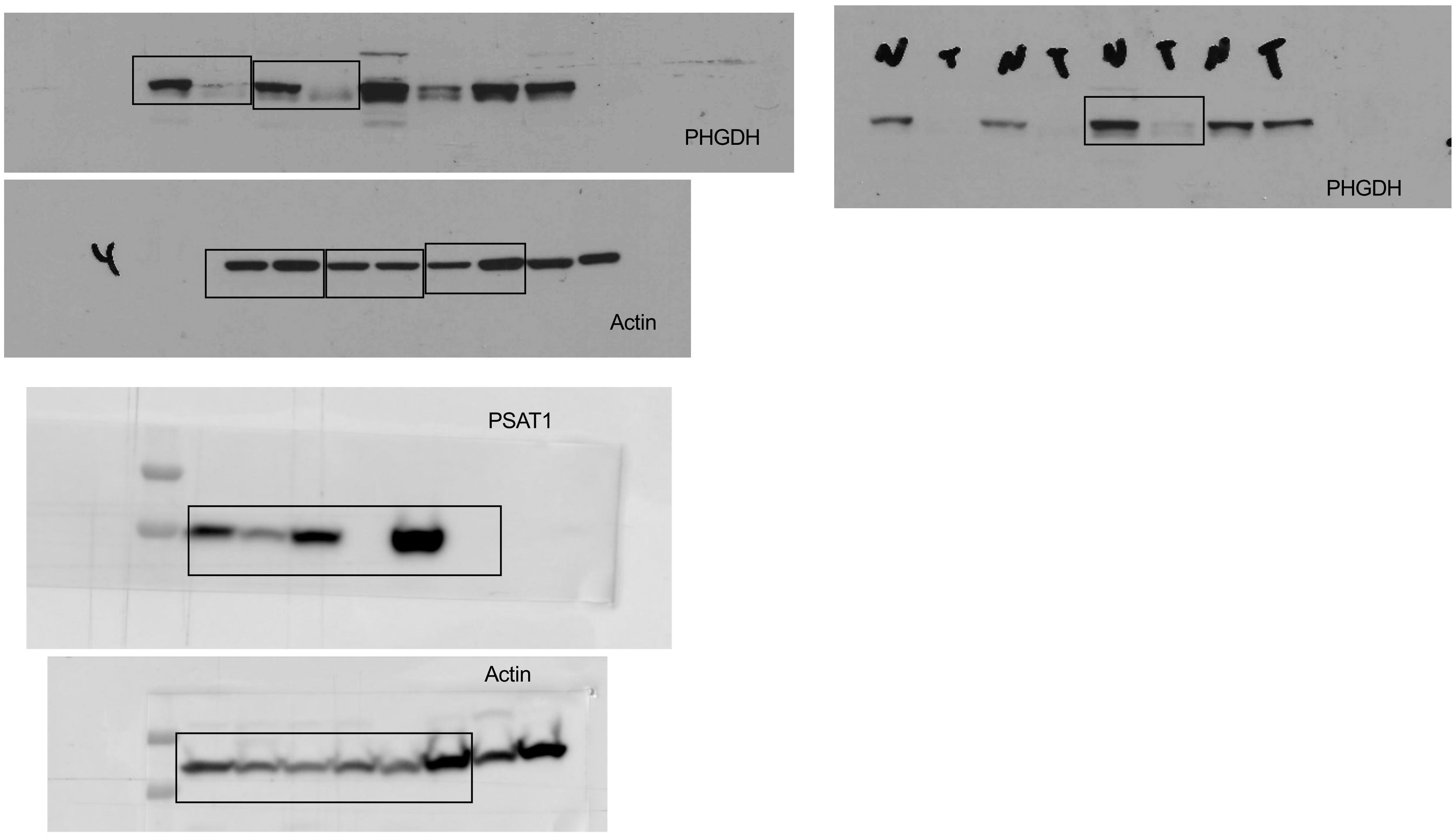

Full unedited gel for Figure 7F

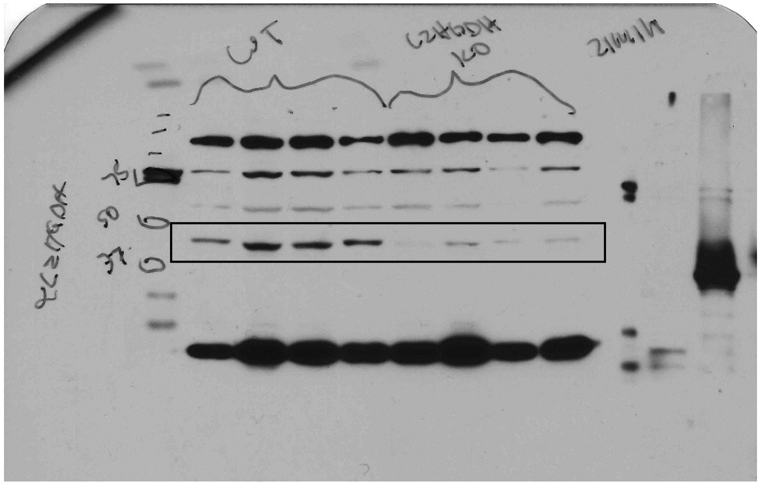

L2HGDH

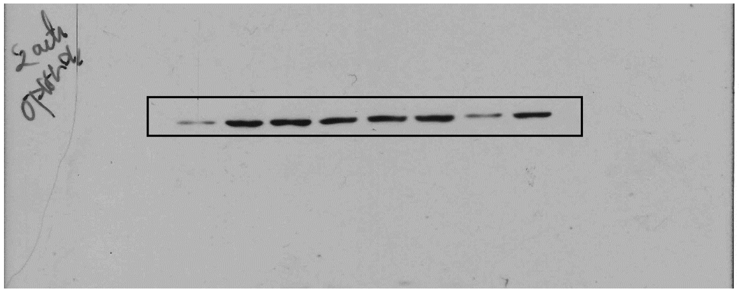

Actin

Full unedited gel for Figure 7H

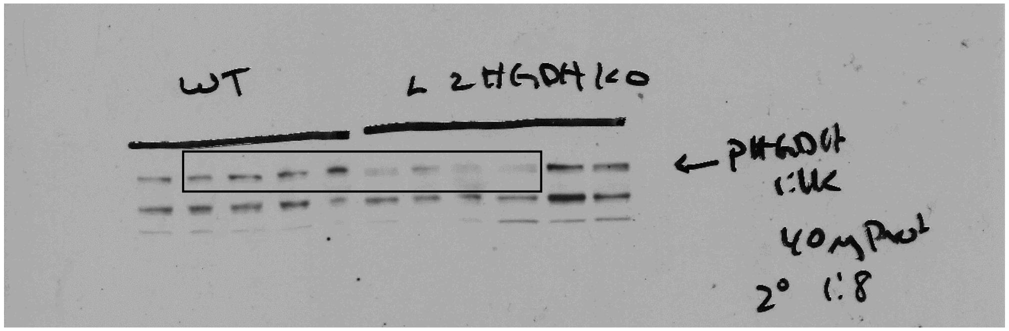

PHGDH

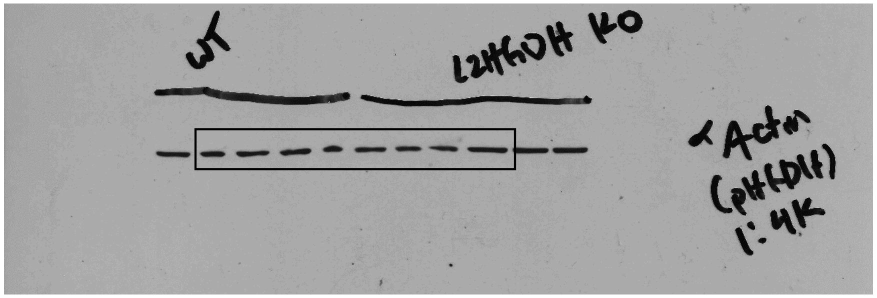

Actin

Full unedited gel for Figure S1D

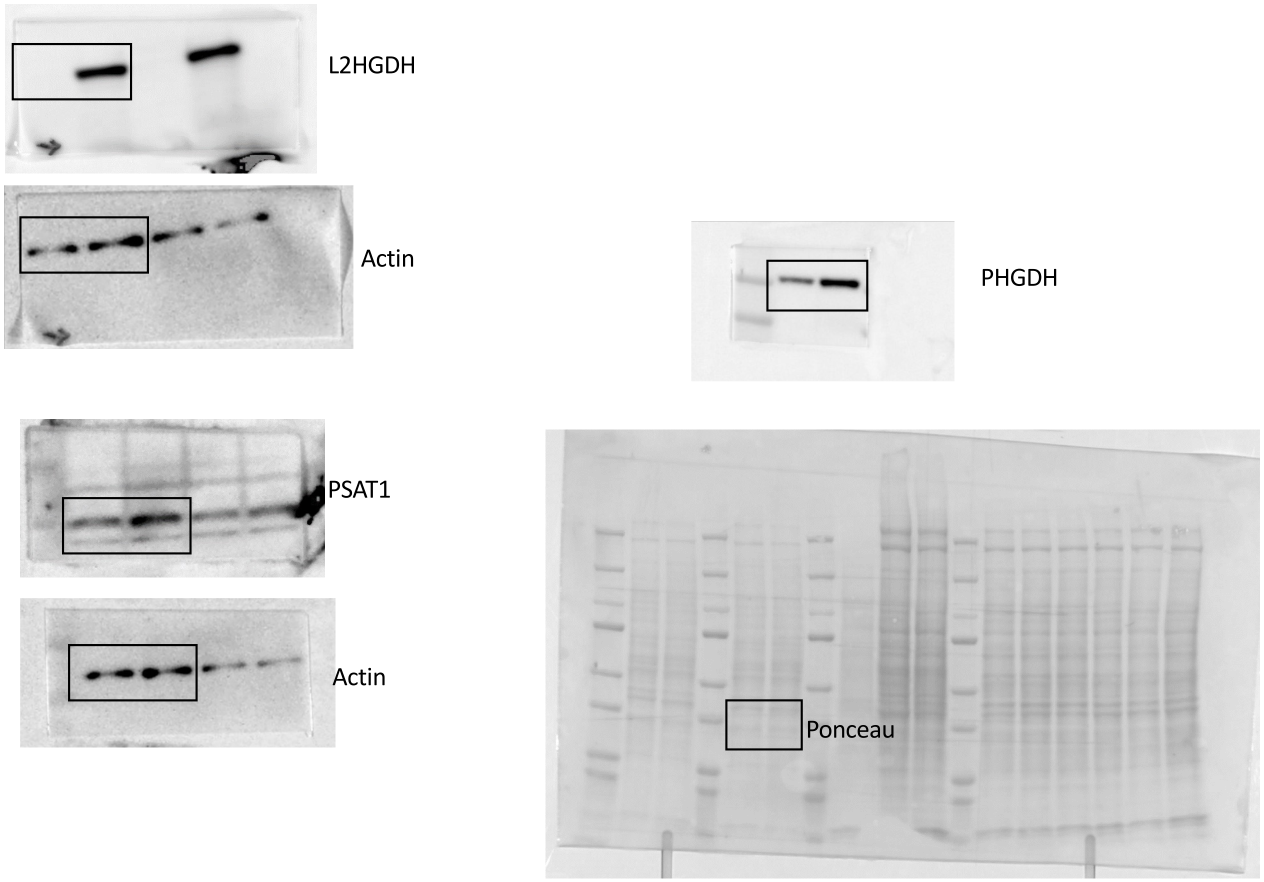

Full unedited gel for Figure S2A

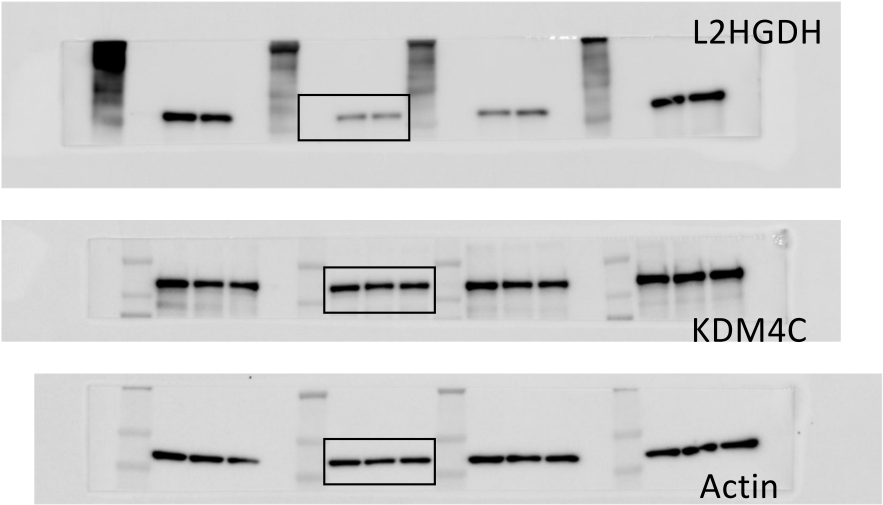

Full unedited gel for Figure S2B

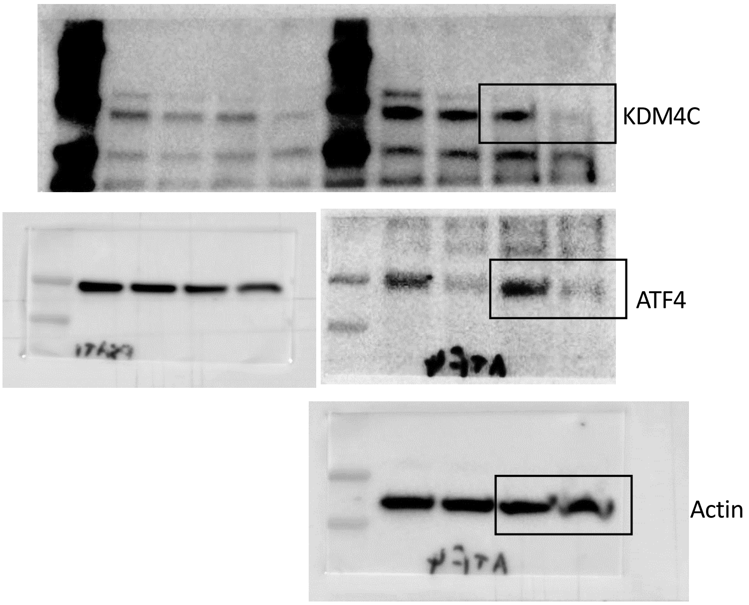

Full unedited gel for Figure S4

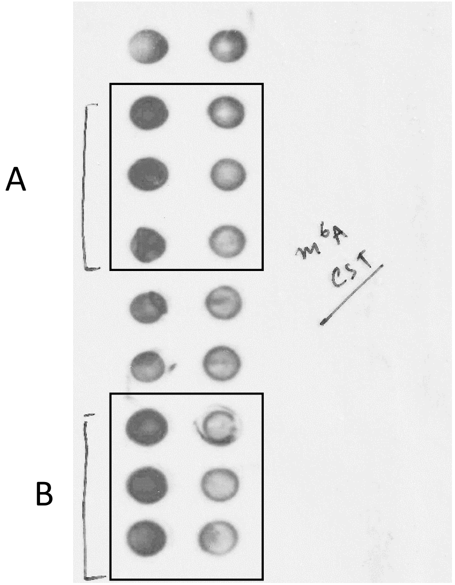

m6A

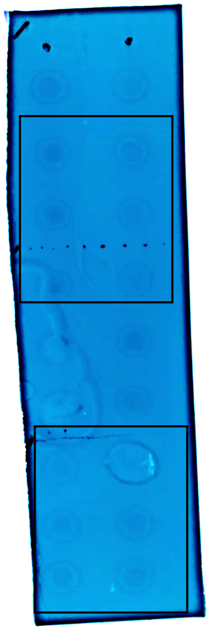

Methylene blue

Uncropped gel for Figure S4E

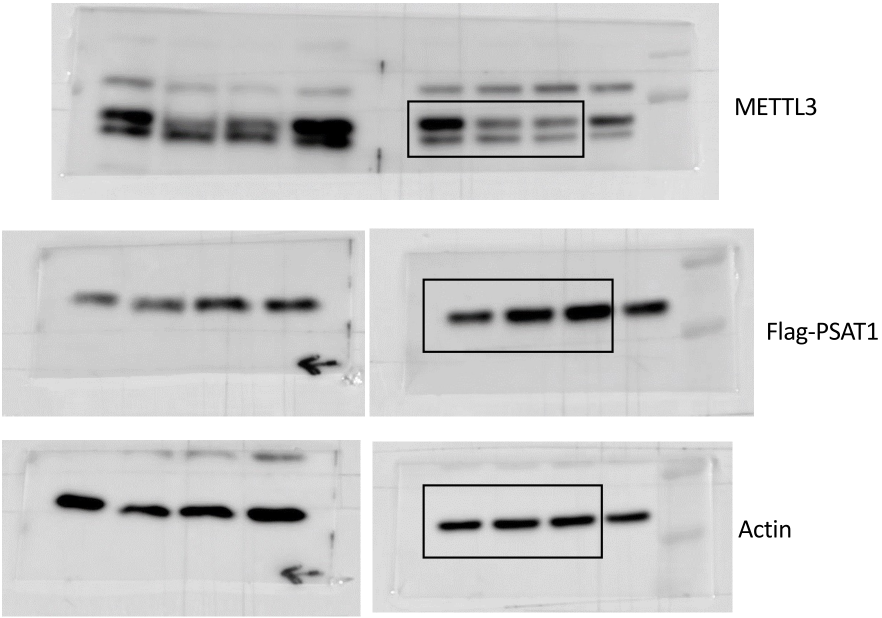

Full unedited gel for Figure S5E

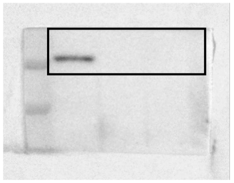

PHGDH

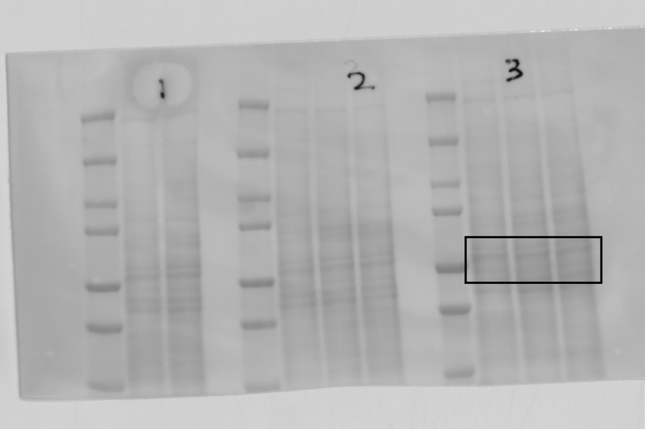

Ponceau

Full unedited gel for Figure S5G

PSAT1

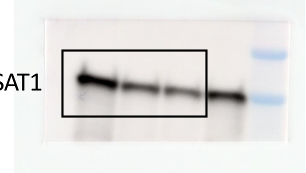

Ponceau

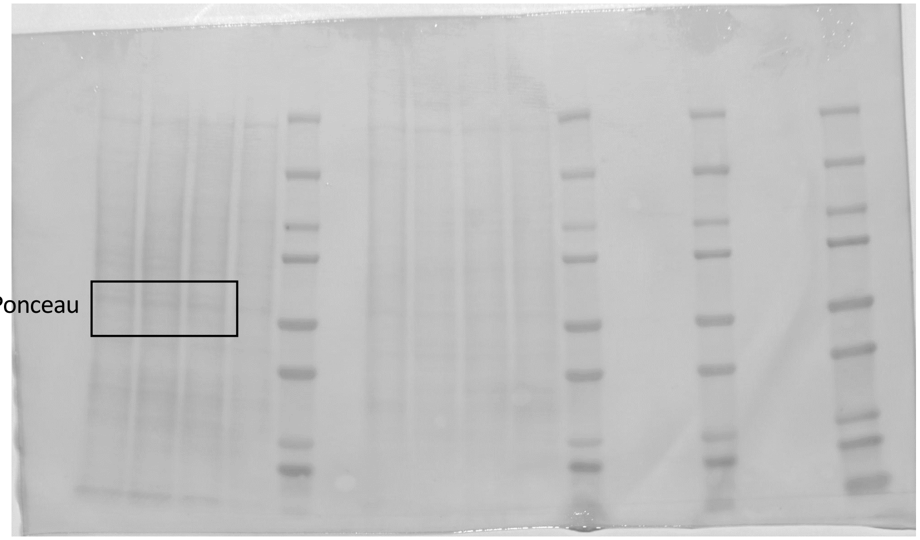

Supplement: Unedited blot and gel images [file jci-134-171294-s016.pdf]
