# Supplementary material for: l-2-Hydroxyglutarate remodeling of the epigenome and epitranscriptome creates a metabolic vulnerability in kidney cancer models
Source: J Clin Invest. 2024 May 14;134(13):e171294. doi: 10.1172/JCI171294 (PMC11213505; doi:10.1172/JCI171294)
Supplement: Supplemental table 5 [file jci-134-171294-s021.pdf]

**Taqman qPCR Probes**	**Company**	**#Catalog**
qPCR probes for PHGDH	Fisher	Hs00198333_m1
qPCR probes for HPRT1	Fisher	Hs02800695_m1
qPCR probes for KDM4C	Fisher	Hs00909577_m1
qPCR probes for ASNS	Fisher	Hs04186194_m1
qPCR probes for PSAT1	Fisher	Hs00795278_mH
qPCR probes for SLC1A5	Fisher	Hs01056542_m1
qPCR probes for SLC1A4	Fisher	Hs00983079_m1
qPCR probes for SLC3A2	Fisher	Hs00374243_m1
qPCR probes for SLC7A5	Fisher	Hs01001183_m1
qPCR probes for SLC38A1	Fisher	Hs01562175_m1
qPCR probes for SLC38A2	Fisher	Hs01089954_m1
qPCR probes for RPLPO	Fisher	Hs99999902 m1
qPCR probes for L2HGDH	Fisher	Hs00227575
qPCR probes for ATF4	Fisher	Hs00909569_g1

**List of siRNAs**	**Company**	**#Catalog**
Scrabled siRNA (Scr)	IDT	51-01-19-09
siALKBH5 (#1)	IDT	hs.Ri.ALKBH5.13.1
siALKBH5 (#2)	IDT	hs.Ri.ALKBH5.13.2
siALKBH5 (#3)	IDT	hs.Ri.ALKBH5.13.3
siFTO (#1)	IDT	hs.Ri.FTO.13.1
siFTO (#2)	IDT	hs.Ri.FTO.13.2
siKDM4C (#1)	IDT	hs.Ri.KDM4C.13.1
siKDM4C (#2)	IDT	hs.Ri.KDM4C.13.2
siMETTL3 (#1)	IDT	hs.Ri.METTL3.13.1
siMETTL3 (#2)	IDT	hs.Ri.METTL3.13.3
On target KDM4C siRNA pool	Dharmacon	L-004293-01-0005

**gRNAs**	**Sequence**
gRNA-3F	5'-CCCGCCCAAACAAGAATTTTCACCG-3'
gRNA-3R	5'-GCTTTCATTAGCATAAGAGCTGCCTCC-3'
Control_gRNA_F (no PAM)	5'-CACCGGTATTACTGATATTGGT-3'
Control_gRNA_R (no PAM)	5'-AAACACCAATATCAGTAATAC-3'

**Primers used in RNA immunoprecipitation-qPCR**	Sequence
PSAT1-3'UTR-F1	5'-CCGCGTATTTTGCCTTTGCT-3'
PSAT1-3'UTR-R1	5'-CCTACCCTCTGTGCTGTGTG-3'

**Primers used in the SELECT Assay**	**Sequence**
SELECT Primer Up	5'-TAGCCAGTACCGTAGTGCGTGTTAATCGCA ACTTGCATTATTAAG-3'
SELECT Primer Down	5'-Phos-CCACAGGCAAGTTTGAAATCCAGAGGCTGAGTCGCTGCAT-3'
M6a-up (-6)	5'-TAGCCAGTACCGTAGTGCGTGCAACTTGCA TTATTAAGTCCACA-3'
M6a-down (-6)	5-Phos GCAAGTTTGAAATCTAACTAGAAAACCAGAGGCTGAGTCGCTGCAT-3'
SELECT qPCR-F	5'-ATGCAGCGACTCAGCCTCTG-3'
SELECT qPCR-R	5'- TAGCCAGTACCGTAGTGCGTG-3'
